# SARS-CoV-2 awakens ancient retroviral genes and the expression of proinflammatory HERV-W envelope protein in COVID-19 patients

**DOI:** 10.1016/j.isci.2023.106604

**Published:** 2023-04-07

**Authors:** Benjamin Charvet, Joanna Brunel, Justine Pierquin, Mathieu Iampietro, Didier Decimo, Nelly Queruel, Alexandre Lucas, María del Mar Encabo-Berzosa, Izaskun Arenaz, Tania Perez Marmolejo, Arturo Ivan Gonzalez, Armando Castorena Maldonado, Cyrille Mathieu, Patrick Küry, Jose Flores-Rivera, Fernanda Torres-Ruiz, Santiago Avila-Rios, Gonzalo Salgado Montes de Oca, Jon Schoorlemmer, Hervé Perron, Branka Horvat

**Affiliations:** 1 GeNeuro Innovation, Lyon, France; 2 CIRI, International Center for Infectiology Research, INSERM U1111, CNRS UMR5308, Université de Lyon, Université Claude Bernard Lyon 1, Ecole Normale Supérieure de Lyon, Lyon, France; 3 We-Met platform, I2MC/Inserm/Université Paul Sabatier UMR1297, Toulouse, France; 4 Biobanco del Sistema de Salud de Aragón, Instituto Aragonés de Ciencias de la Salud (IACS), Zaragoza, Spain; 5 Instituto Nacional de Enfermedades Respiratorias Ismael Cosio Villegas, México Ciudad, México; 6 Department of Neurology, Medical Faculty, Heinrich-Heine-University, Dusseldorf, Germany; 7 Department of Neurology, National Institute of Neurology and Neurosurgery, Mexico City, Mexico; 8 Centro de investigación en Enfermedades Infecciosas, Instituto Nacional de Enfermedades Respiratorias Ismael Cosío Villegas, México Ciudad, México; 9 ARAID Fundación; Instituto Aragonés de Ciencias de la Salud (IACS); Grupo B46_20R de la DGA and GIIS-028 del IISA; all Zaragoza, Spain; 10 GeNeuro, Plan les Ouates, Geneva, Switzerland

**Keywords:** Health sciences, Virology, Molecular Genetics

## Abstract

Patients with COVID-19 may develop abnormal inflammatory response, followed in some cases by severe disease and long-lasting syndromes. We show here that *in vitro* exposure to SARS-CoV-2 activates the expression of the human endogenous retrovirus (HERV) HERV-W proinflammatory envelope protein (ENV) in peripheral blood mononuclear cells from a subset of healthy donors, in ACE2 receptor and infection-independent manner. Plasma and/or sera of 221 COVID-19 patients from different cohorts, infected with successive SARS-CoV-2 variants including the Omicron, had detectable HERV-W ENV, which correlated with ENV expression in T lymphocytes and peaked with the disease severity. HERV-W ENV was also found in postmortem tissues of lungs, heart, gastrointestinal tract, brain olfactory bulb, and nasal mucosa from COVID-19 patients. Altogether, these results demonstrate that SARS-CoV-2 could induce HERV-W envelope protein expression and suggest its involvement in the immunopathogenesis of certain COVID-19-associated syndromes and thereby its relevance in the development of personalized treatment of patients.

## Highlights

• SARS-CoV-2 induces endogenous retrovirus envelope in a subset of healthy donors
• HERV-W ENV is detected on lymphocytes, in serum and tissues of COVID-19 patients
• HERV-W ENV protein in serum and lymphocytes correlates with COVID-19 severity
• Prognostic value of HERV-W ENV expression may allow targeted therapy of COVID-19

## Graphical abstract

**Figure:**
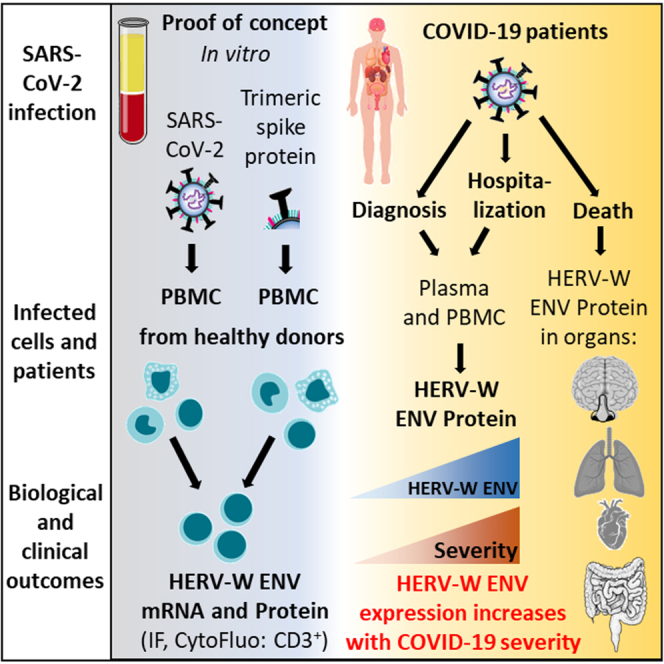

## Introduction

The human “coronavirus disease 2019” (COVID-19) caused by the “severe acute respiratory syndrome coronavirus 2” (SARS-CoV-2) continues to cause high morbidity and mortality, with uncertain but recurrent evolution with the emergence of viral variants.^1^ Numerous long-lasting, post-infectious symptoms or syndromes are commonly observed among patients who had COVID-19, from benign to severe forms.^2^ Moreover, beyond a dominant respiratory tract tropism, extra-pulmonary COVID-19 forms are more frequent and diverse than initially expected.^3^

The dysregulation of innate and adaptive immunity has been recognized to play a critical role in the clinical outcome of COVID-19 patients. Severe evolution of COVID-19 is thought to be driven by hyperactivated innate immunity,^4^ in addition to adaptive immune defects often resulting in lymphopenia and neutrophils/lymphocytes imbalance.^5^ A deficient interferon response has also been shown to favor or result from SARS-CoV-2 infection.^6^^,^^7^ Multifaceted immunological dysregulations are underlying hyper-immune reactions such as the “cytokine storm” syndrome, the multisystem inflammatory syndrome in children, and inflammation-driven thromboembolic events, as well as neurological and various other manifestations.^7^^,^^8^^,^^9^^,^^10^ The present COVID-19 pandemic has thus raised many questions about the pathophysiological mechanisms that could explain numerous symptoms and syndromes associated with SARS-CoV-2 infection.

Certain infectious agents have been shown to activate pathological processes via receptor-coupled signaling pathways, by impairing the epigenetic control and/or by directly activating endogenous retroviral elements (human endogenous retroviruses [HERVs]) present in the human genome.^11^ HERVs represent about 8% of human chromosomal sequences and comprise about 22 families independently acquired during evolution from exogenous retroviruses via an infection of germline cells.^12^^,^^13^ In particular conditions of activation, a production of endogenous proteins of retroviral origin with pathogenic effects may generate clinical symptoms corresponding to the organ, tissue, or cells in which they are expressed according to the specific tropism of the triggering infectious agent.^14^^,^^15^^,^^16^ HERV abnormal expression may also become self-sustained, thus creating chronic protein expression in affected tissues, e.g., with cytokine-mediated feedback loops.^17^ Such a sustained expression has been shown to be involved in brain lesions with lifelong expansion in patients with multiple sclerosis (MS).^15^^,^^18^^,^^19^ HERV envelope proteins (ENVs) can be inserted in cell membranes but may also be released extracellularly. Some of them were shown to exert major immunopathogenic^20^^,^^21^^,^^22^^,^^23^ and/or neuropathogenic^19^^,^^24^ effects *in vitro* and *in vivo*, associated with disease pathognomonic features.

We therefore studied whether SARS-CoV-2 could activate HERV copies considered as “dormant enemies within”.^25^ We focused on HERV families already shown to be involved in the pathogenesis of human diseases, HERV-W and HERV-K,^26^ to comprehensively evaluate their potential association with COVID-19 and associated syndromes. This question became critical after a recent study has revealed the significant expression of HERV-W ENV in lymphoid cells from COVID-19 patients, correlating with disease outcome and markers of lymphocyte exhaustion or senescence.^27^

In the present study, we initially addressed the potential role of SARS-CoV-2 to activate a pathogenic HERV protein expression, as reported with other viruses in, e.g., MS and in type 1 diabetes.^11^^,^^16^^,^^28^ We further analyzed their expression in white blood cells and the presence in plasma of patients with COVID-19 presenting various clinical forms at early and late time points. Our results showed that (i) SARS-CoV-2 can activate the production of HERV-W ENV in cultured blood mononuclear cells from a subset of healthy donors, (ii) HERV-W ENV is expressed on T lymphocytes from COVID-19 patients, (iii) HERV-W ENV antigen is detected in all tested plasma or serum samples from severe cases in intensive care unit but only in about 20% of PCR-positive cases after early diagnosis, (iv) the prevalence of HERV-W ENV increases with disease severity whatever the infecting SARS-CoV-2 variant is implicated, and (iv) HERV-W ENV expression is observed by immunohistochemistry in cell types relevant for COVID-19-associated pathogenesis within affected organs and, particularly, in microglia of postmortem brain parenchyma from severe COVID-19 patients. Altogether, these results strongly suggest that HERV-W ENV may be involved in immunopathogenic pathways associated with acute and post-acute COVID, underlying the importance to further address its role as a biomarker and as a potential target for personalized treatment of COVID-19 patients.

## Results

### SARS-CoV-2 triggers HERV-W and HERV-K ENV mRNA early transcription along with HERV-W ENV protein in peripheral blood mononuclear cells (PBMCs) of healthy donors

**Figure 1:**
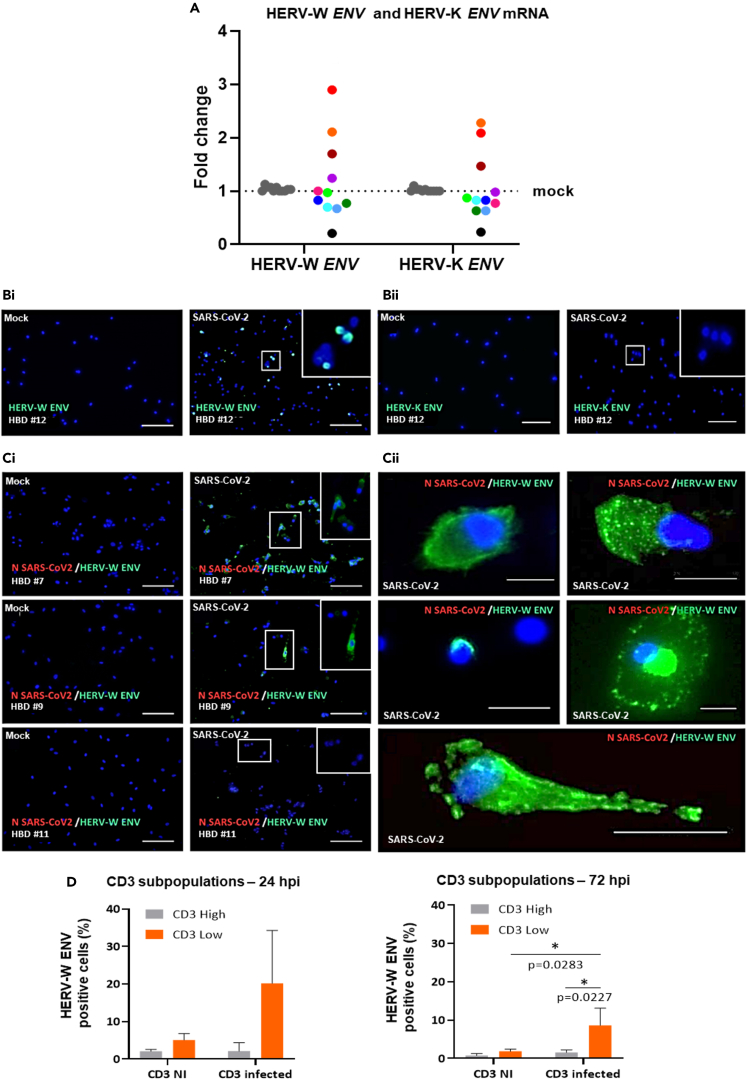
Expression of HERV-W *ENV* or HERV-K *ENV* RNA and HERV-W ENV protein in PBMC from healthy blood donors (HBDs) exposed to SARS-CoV-2 *in vitro* (A) The level of HERV-W ENV and HERV-K *ENV* mRNA in PBMC cultures from HBD, exposed to SARS-CoV-2 (MOI:0.1) or mock-treated (culture medium), was analyzed by RT-qPCR. The graph presents the mean results from triplicate (7 out 11 donors) or single cultures (4 out 11 donors) at 2h post-exposure. For each healthy blood donor, a color was assigned in order to be able to compare the induction of HERV-W ENV and HERV-K ENV according to the cultures of PBMC from the same individual. The details of the RT-qPCR results are available on the following dataset: https://doi.org/10.17632/rc74sdgksk.1. (B and C) PBMC cultures of 8 HBD, inoculated or not with SARS-CoV-2 at 0.1 MOI, were collected either 3 or 7 days post-exposure and stained for HERV-W ENV (green) and anti-SARS-CoV-2 nucleocapsid (red). (B) PBMCs from HBD #12 were inoculated or not with SARS-CoV-2 (MOI: 0.1). Bi: HERV-W ENV labeling, Bii: HERV-K ENV labeling (green staining). (C) PBMC cultures from 3 representative donors with variable number of HERV-W ENV-positive cells (green staining): donor #7 at day 7 and donor #9 at day 3 and a culture form a non-responding donor #11 at day 7 (Ci). The different morphological aspects of HERV-W ENV-positive cells are presented with high magnifications (Cii). DAPI was used to stain nuclei (blue staining). Bars B-F’ = 100 μm; Bars G-K: 25 μm. (D) PBMCs from healthy donors were either incubated with SARS-CoV-2 (MOI = 0.1) for 24 h or 72 h or remained unexposed to the virus. Cells were stained using anti-CD14, anti-CD3, and GN_mAb_Env01 antibodies and analyzed by flow cytometry (Figure S1). The percentage of HERV-W ENV-positive cells within CD3^high^ and CD3^low^ T cell sub-populations was determined by cytofluorometry and presented with histograms (mean from 3 donors ±SD). NI: mock-infected cells. Statistical analysis was performed as described in STAR Methods (∗p < 0.05, ∗∗p < 0.01, ∗∗∗p < 0.001 and ∗∗∗∗p < 0.0001).

We initially analyzed whether infectious SARS-CoV-2 could modulate the expression of HERV-W and HERV-K *ENV* genes in leukocytes from healthy blood donors (HBDs). PBMCs were cultured with or without infectious SARS-CoV-2, and RNA was collected at 2h post-inoculation. In PBMC from 3 out of 11 HBDs (27%), HERV-W *ENV* RNA levels were significantly increased after exposure to SARS-CoV-2 (Figure 1A). The same donors also showed a transcriptional increase of HERV-K *ENV* while in some donors the decrease of the expression of both ENVs was observed. The comparison of average fold changes for “HERV-activating” samples (1.99 ± 0.7 for W-*ENV* and 1.95 ± 0.4 for K-*ENV*) and “non HERV-activating” subgroup (0.74 ± 0.26 for W-*ENV* and 1.06 ± 0.23 for K-*ENV*) showed consistently highly significant difference, (i) an increase in mRNA expression in virus-exposed versus mock-exposed cells for both W-*ENV* and K-*ENV* in the subgroup of HERV-activating donors and (ii) decrease in RNA expression in virus-exposed versus mock-exposed cells for W-*ENV* and K-*ENV,* in the subgroup of HERV-non-activating donors (p < 0.0001, F test). Interestingly, the cells from the same donor presented the similar pattern of the expression for both W-*ENV* and K-*ENV,* depending on the subgroup they belonged. Of note the baseline expression of HERV-W and HERV-K copies co-amplified by the same primers is due to highly homologous sequences in several other copies from the same families, whatever the primers used. In control cultures, this reflects the detection of RNAs from these HERV families, now globally known to contribute to non-coding regulatory RNAs involved in physiological controls of gene expression.^29^^,^^30^^,^^31^ Thus, the inhibitory effect impacting both HERV-W and HERV-K RNA, as observed in certain donors, is consistent with molecules and pathways targeting retrovirus-specific sequence motifs.^32^^,^^33^^,^^34^

We next analyzed whether HERV RNA expression was followed by HERV protein production. PBMC cultures of independent HBD, inoculated with SARS-CoV-2 or mock-inoculated, were analyzed for the presence of envelope proteins of HERV-W, HERV-K, or SARS-CoV-2 N by immunofluorescence (IF) (Figures 1B and 1C). HERV-W ENV expression was donor dependent as observed in 3 out of 8 HBDs PBMC cultures inoculated with SARS-CoV-2. It was abundantly detected in donor #7, moderately in #12, and in isolated cells of #9, whereas it was not detected in others like donor #11 (Figures 1B and 1C). Nevertheless, in HERV-W ENV-positive cells of “responders”, a marked cellular expression was seen at high magnification (Figure 1C). Conversely, neither HERV-K ENV (Figure 1Bi) nor SARS-CoV-2 antigens (Figures 1B and 1C) were detected in any of the analyzed conditions. Finally, neither HERV-W ENV nor HERV-K ENV protein was detected in control cultures without exposure to SARS-CoV-2 (Figures 1B and 1C). In SARS-CoV-2-infected Vero E6 cells, used as a positive control, a clear cellular expression of SARS-CoV-2 N and spike proteins (Figure S1A), along with an increased SARS-CoV-2 *N* mRNA load, was confirmed (Figure S1B). The specificity of the anti-HERV-W and -K ENV antibodies had been established in previous studies,^15^^,^^35^ and that of secondary antibodies was also verified on PBMC from studied HBD (Figure S1C).

To compare HERV RNA kinetics in PBMC exposed to SARS-CoV-2 from different individuals, the PBMCs of 4 representative HBDs were analyzed (Figure S1D). Results showed that the levels of both HERV-W *ENV* and HERV-K *ENV* RNA decreased at 19h and/or 24h post-inoculation after an earlier peak in cells from “HERV-activating” donors. In parallel, in the same cell cultures, SARS-CoV-2 *N* RNA kinetics showed abundant RNA load only after viral inoculation in all inoculated samples, with a significant decrease at 19h post-exposure, confirming the absence of viral replication in PBMC (Figure S1E).

### Exposure to SARS-CoV-2 triggers HERV-W envelope production in a T cell subset of healthy individuals

HERV-W ENV protein expression has been previously observed in CD3^+^ T cells of COVID-19 patients.^27^ We therefore analyzed whether direct exposure to SARS-CoV-2 could induce HERV-W ENV in T lymphocytes from healthy donors as well. For this purpose, HERV-W ENV expression in PBMC cultures from HBD, with or without exposure to SARS-CoV-2, was analyzed using cytofluorometry analysis on permeabilized cells. As illustrated in Figure S2A, the gating strategy identified CD3^+^ T lymphocytes in non-infected (NI) cultures. CD3^+^ cells comprised CD3^high^ T cells, physiologically representing naive/non-activated cells, and CD3^+^ cells with increased size, normally representing activated T cells, further decreasing CD3 level at their surface and corresponding to the CD3^low^ subpopulation.^36^^,^^37^ To discriminate CD14^+^ monocytes from larger CD3^low^ T cells, we eliminated CD14^+^ cells by specifically gating CD14^−^ cells. After exposure to SARS-CoV-2, CD3^+^ cells showed HERV-W ENV cell surface expression. Double labeling with anti-CD3 and anti-HERV-W ENV antibodies revealed that a significant proportion of CD3^low^ T cells were positive for HERV-W ENV both at 24h and 72h after exposure to SARS-CoV-2 (Figures 1D and S2A, CD3^+^ top panels), compared to CD3^high^ T cells (Figures 1D and S2A; CD3^+^ bottom panels), while mock-infected cultures were negative (Figures 1D and S1A, CD3^+^ top and bottom panels). Globally, minimal fluorescence associated with HERV-W ENV detection (compatible with technical background noise) was observed with CD3^+^ T cells from mock-inoculated PBMC, whereas a significant increase was characterized in SARS-CoV-2-exposed PBMC and mainly observed in CD3^low^ T cells (Figure 1D).

### Exposure to SARS-CoV-2 recombinant trimeric spike protein triggers HERV-W ENV protein production in PBMC in a subgroup of heathy individuals

The very rapid response to SARS-CoV-2 virus characterized by an early peak of RNA followed by HERV-W ENV protein expression in PBMC (Figures 1A and S1D), in the absence of detectable infection by SARS-CoV-2, prompted us to investigate a possible direct stimulation by SARS-CoV-2 proteins, particularly by its surface spike protein, similarly to what had been shown for human herpes virus type 6 (HHV-6) in glial cells.^38^

**Figure 2:**
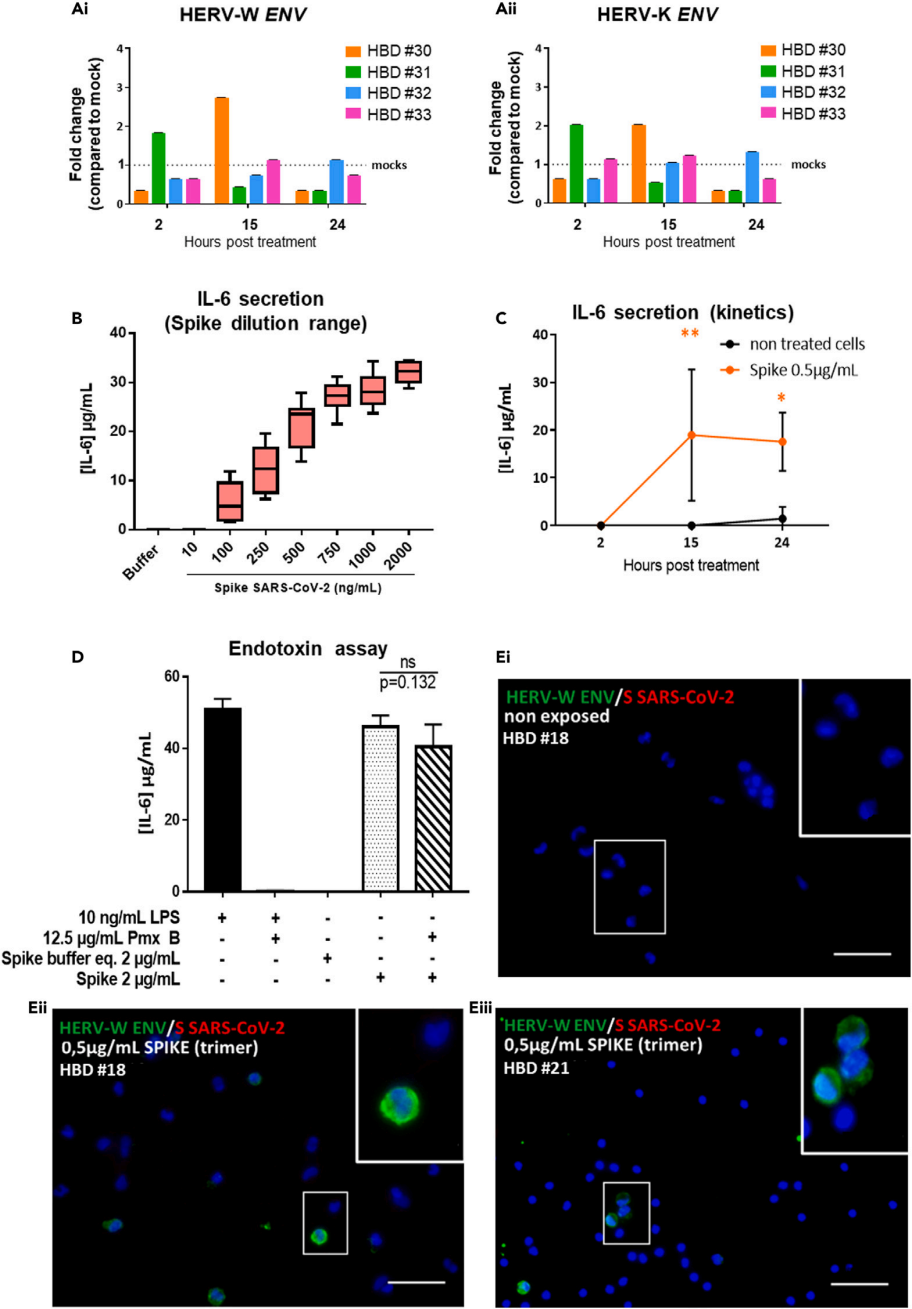
Induction of HERV-W *ENV* and HERV-K *ENV* mRNA after exposure to SARS-CoV-2 spike trimer, followed by HERV-W ENV protein expression in PBMC from healthy blood donors (A) PBMCs from 4 HBDs were exposed to 0.5 μg/mL of active trimer spike recombinant protein in parallel to mock control (buffer), and HERV-W *ENV* (Ai) and HERV-K *ENV* (Aii) mRNA levels were assessed at 2, 15, and 24h post-inoculation (pi) by RT-qPCR. (B) PBMCs isolated from HBD were incubated or not with recombinant spike trimer. IL-6 secretion was monitored at 24h by ELISA in cultures inoculated with increasing concentrations of non-stabilized SARS-COV-2 spike protein. (C) Kinetics of IL-6 release at 2, 15, and 24h post treatment (hpt) with 500 ng of spike protein or without was performed with 3 donors (#30–32). (D) To exclude an effect of endotoxin possibly present in the recombinant protein buffer, the PBMCs of 3 HBDs were exposed either to 10 ng/mL of LPS or to 2 μg/mL of recombinant spike, in combination or not with 12.5 μg/mL of polymyxin B (PMB). IL-6 was then quantified in the culture supernatants by ELISA. The volume of buffer equivalent to 2 μg/mL of spike protein was used as a negative control. (E) PBMCs from healthy blood donors were exposed during 24h with 0.5 μg/mL of recombinant spike trimer. Results obtained on 2 responding donors [(# 18 (Eii) and #21 (Eiii)] are presented. Untreated PBMCs from the responding donor #18 (Ei). (Ei-Eii) HERV-W ENV was detected in few cells of cultures exposed to the spike trimer (geen staining). Higher magnifications of HERV-W ENV-positive cells are presented in white squares. DAPI was used to stain nuclei (in blue). Bars c-e: 50 μm. Statistical analysis was performed as described in STAR Methods (∗p < 0.05, ∗∗p < 0.01, ∗∗∗p < 0.001 and ∗∗∗∗p < 0.0001).

A recombinant trimeric spike protein corresponding to the spike expressed on initially circulating SARS-CoV-2 isolate^39^ was added into the culture medium of PBMC from HBD (Figure 2). PBMC RNA was collected at 2h, 15h, and 24h post-inoculation and analyzed by RT-qPCR for HERV-W and HERV-K envelope gene expression. Donor #31 showed increased RNA levels for both HERV-W and HERV-K *ENV* at 2h post-inoculation; those from donor #30 peaked at 15h, while RNA levels from both donors #32 and #33 immediately (≥2h) dropped below the baseline of non-exposed cells (Figure 2A), a pattern similar to the one observed in HBD PBMC “non-responding” to infectious SARS-CoV-2 (Figure S1D). Interestingly, donor #30 was the only one tested positive for anti-SARS-CoV-2 serum antibodies, which did not prevent HERV transcriptional activation by this spike trimer but coincided with a delayed peak of HERV RNA.

We next analyzed whether the exposure to recombinant spike could induce interleukin 6 (IL-6) secretion from PBMC from HBDs. Initially, we tested a large range of concentrations and found that the induction of IL-6 was observed from 100 ng of spike protein in all tested donors (Figure 2B). IL-6 kinetics analysis was performed on HBD PBMC tested for HERV expression using 500 ng of the spike protein. Results showed a significantly increased IL-6 production starting from 15h after exposure in PBMC culture supernatants of all donors, including HERV “non-responding” ones (Figure 2C). IL-6 production was not linked with possible endotoxin contamination as the treatment with the endotoxin inhibitor polymyxin B did not diminish the spike-induced IL-6 secretion (Figure 2D). Thus, data demonstrated that spike-induced HERV activation occurs very early after exposure, before the release of IL-6 and only in a subgroup of healthy PBMC donors, therefore independently from IL-6 production.

HERV-W ENV protein production was confirmed by IF analysis at 72h in cultured PBMC of two donors inoculated with spike trimers (500 ng/mL) and not in mock-control cultures (Figure 2E). HERV-W ENV-positive cells were detected as previously seen with the infectious virus. Interestingly, the absence of SARS-CoV-2 entry receptor (angiotensin converting enzyme-2, ACE2) expression in PBMC (Figure S2B) suggests an interaction of the spike protein with another receptor. Finally, results were not influenced by the cell viability, which did not vary significantly within the analyzed culture period (≤5 days; Figure S2C).

### HERV-W ENV protein is expressed at the surface of T lymphocytes from COVID-19 patients and correlates with the detection of soluble HERV-W ENV hexameric protein in plasma

**Figure 3:**
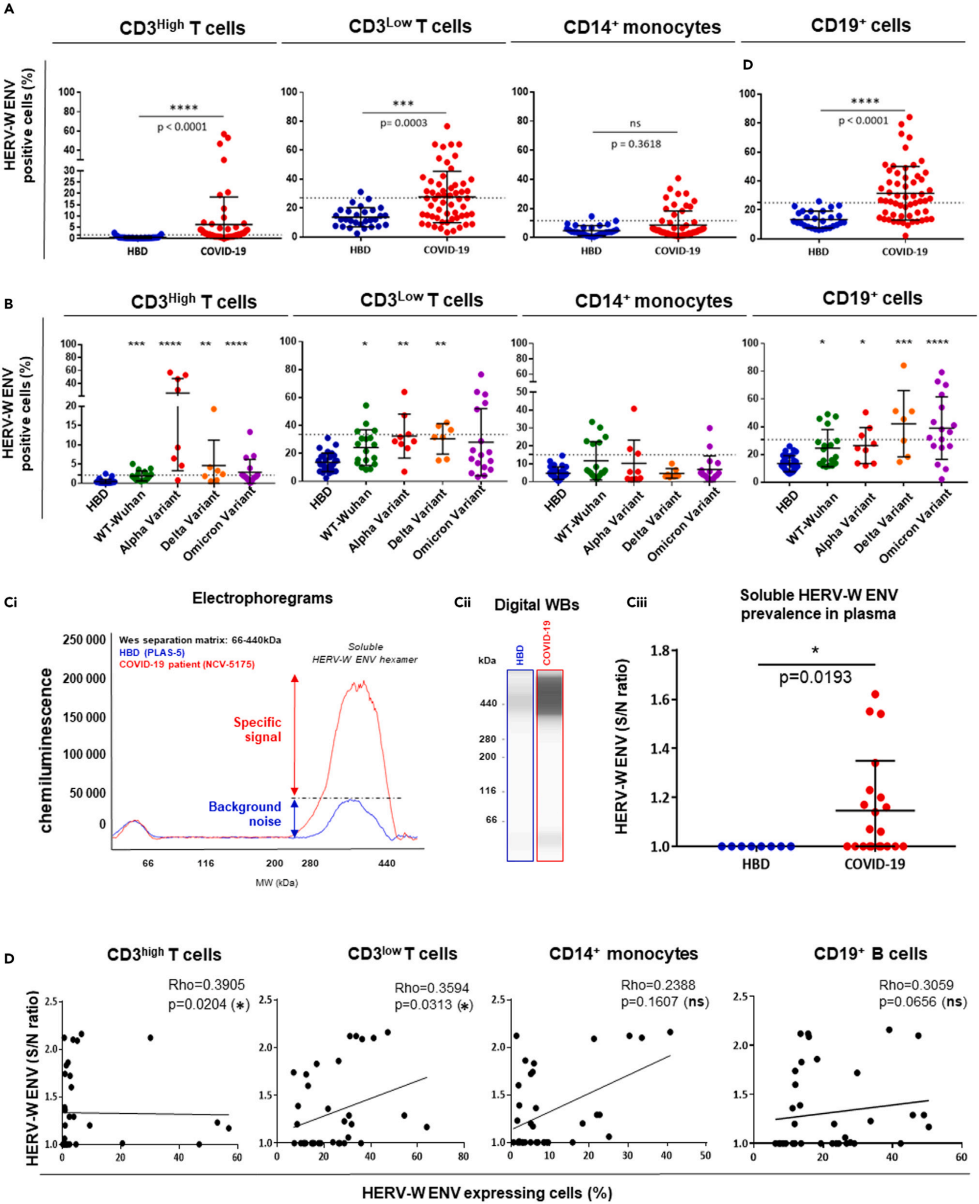
COVID-19 patients produce HERV-W ENV in the plasma and on the membrane of their PBMC (A) The percentage of HERV-W ENV-positive cells was analyzed by cytofluorometry in PBMC from available fresh PBMC of 33 healthy blood donors (HBDs) (blue dots) and 57 COVID-19 patients (red dots) hospitalized in Lyon. The percentage of HERV-W ENV-positive cells has been evaluated in CD3high T cells, CD3low T cells, CD14+ monocytes, and CD19^+^ B cells. The dotted lines correspond to the positivity threshold calculated from the mean of HBD values +3SD. (B) Distribution of patients of the same cohort infected with the different SARS-CoV-2 variants. The dotted lines correspond to the positivity threshold calculated from the mean of HBD values +3SD. (C) Electrophoregrams (Ci) and digital western blot representations -WBs- (Cii) from healthy blood donors (HBDs, blue panel) and COVID-19 patients (red panel). Dotted line in Ci represents the upper limit of non-specific chemiluminescence due to the post-migration deposit of high molecular weight protein trapping detection reagents. This background signal is also seen on “HBD” run of the digital WB representation. (Ciii) Quantification of the soluble HERV-W ENV hexamer in the plasma of 8 HBDs (blue dots) and 21 COVID-19 patients (red dots) from Lyon series with available plasma samples (cf. clinical data of Lyon cohort; https://doi.org/10.17632/3v4hfxv4w8.1). (D) Scatterplots presenting the correlation between quantification of the soluble HERV-W ENV in plasma (Y axis) and the percentage of HERV-W ENV-positive cells in the different PBMC sub-populations (X axis) in blood of 14 HBDs and 27 COVID-19 patients from “Lyon cohort” with available plasma and PBMC samples (cf. clinical data of Lyon cohort; https://doi.org/10.17632/3v4hfxv4w8.1). The gating strategy for cytofluorometry analyses is presented in Figure S2D. Statistical analysis was performed as described in STAR Methods (∗p < 0.05, ∗∗p < 0.01, ∗∗∗p < 0.001 and ∗∗∗∗p < 0.0001).

We next analyzed the levels of HERV-W ENV expressed on the membrane of PBMC from hospitalized COVID-19 patients and compared them with the detection of the soluble protein in plasma samples available in parallel from a recruitment series of 21 patients (Figure 3, patients #28 to #48). Biological and clinical data are presented in https://doi.org/10.17632/3v4hfxv4w8.1.

The proportion of HERV-W ENV-positive cells was analyzed in PBMCs from 57 patients with COVID-19 (patients #1 to #27 and #192 to #221) compared to 31 HBDs (Figure 3A). As for previous analyses, the gating strategy selected CD14^−^ cells to specifically analyze CD3^+^ T lymphocytes and CD14^+^ to investigate monocytes (Figure S2D). A highly significant difference of HERV-W ENV expression on CD3^+^ T cells was observed between COVID-19 patients and HBD controls. The percentage of HERV-W ENV-positive CD3^high^ T cells was low but significantly higher when compared to HBD (p < 0.0001). HERV-W ENV-positive CD3^low^ T cells of COVID-19 individuals were numerous and significantly elevated compared to HBD (p = 0.0003). Interestingly, while no difference between HBD and COVID-19 patients was observed in CD14^+^ monocytes stained for HERV-W ENV, a significant expression was also observed in CD19^+^ B cells from COVID-19 patients, compared to HBD (p < 0.0001) (Figure 3A).

The same PBMCs from four series of patients recruited during pandemic waves with successive variants were next studied in the cohort of 51 COVID-19 patients (patients #1 to #27 and #192 to #221 excepted patients without variant identification cf. “Lyon” cohort clinical data; https://doi.org/10.17632/3v4hfxv4w8.1) split into subgroups corresponding to infections with Wuhan, Alpha, Delta, and Omicron variants of SARS-CoV-2 (Figure 3B). In all cases, regardless of the SARS-CoV-2 variant, CD3^+^/HERV-W ENV^+^ T lymphocytes were significantly detected in COVID-19 patients, i.e., values above the threshold of the background signal noise varying with cell type, represented by the “mean+2SD” of values from HBD, homogeneously distributed and grouped below this threshold. However, despite a significant difference on CD3^high^/HERV-W ENV^+^ cells, a lower proportion had CD3^low^/HERV-W ENV^+^ lymphocytes among the patients infected with the Omicron variant. The Omicron series included patients hospitalized with less severe forms, whereas most patients with other variants were admitted into intensive care units. Interestingly, patients infected with Omicron and Delta variants presented the highest and most significant proportion of CD19^+^/HERV-W ENV^+^ B lymphocytes.

HERV-W ENV soluble protein, which presents a hexameric structure,^15^ was further quantified by immunocapillary analysis in the plasma of 8 HBDs and 21 COVID-19 patients (patients #28 to #48 from “Lyon” cohort described in https://doi.org/10.17632/3v4hfxv4w8.1) presenting different levels of severity. The prevalence of HERV-W ENV soluble protein was significantly elevated in plasma from COVID-19 patients (11/21), compared to HBDs (0/8) (Figure 3C). Conversely, HERV-K ENV antigen was not detected in any plasma sample (Figure S3A). As shown in Figure S3C, the specificity of these detections was also validated with an isotype control antibody, which gave non-detectable signal in plasma samples from COVID-19 patients.

The levels of HERV-W ENV soluble hexameric protein in plasma of COVID-19 patients correlated with the percentage of HERV-W ENV-positive T-CD3^+^ cells, as determined by cytofluorometry in the same blood samples (patients #1 to #27 and 8 HBD) (Figure 3D). The correlation observed between HERV-W ENV released in plasma and its expression on the membrane of both CD3^high^ and CD3^low^ T lymphocytes was statistically significant, while no correlation was found for CD14^+^ monocytes and CD19^+^ B cells. Thus, in addition to the previously reported correlation between HERV-W ENV expression quantified by RT-qPCR and the expression determined by cytofluorometry in CD3^+^ T lymphocytes from COVID-19 patients,^27^ these results provide another orthogonal validation of HERV-W ENV protein detection by two unrelated techniques in COVID-19 blood samples.

### The prevalence of soluble HERV-W ENV in plasma from COVID-19 patients correlates with blood biomarkers and disease severity

**Figure 4:**
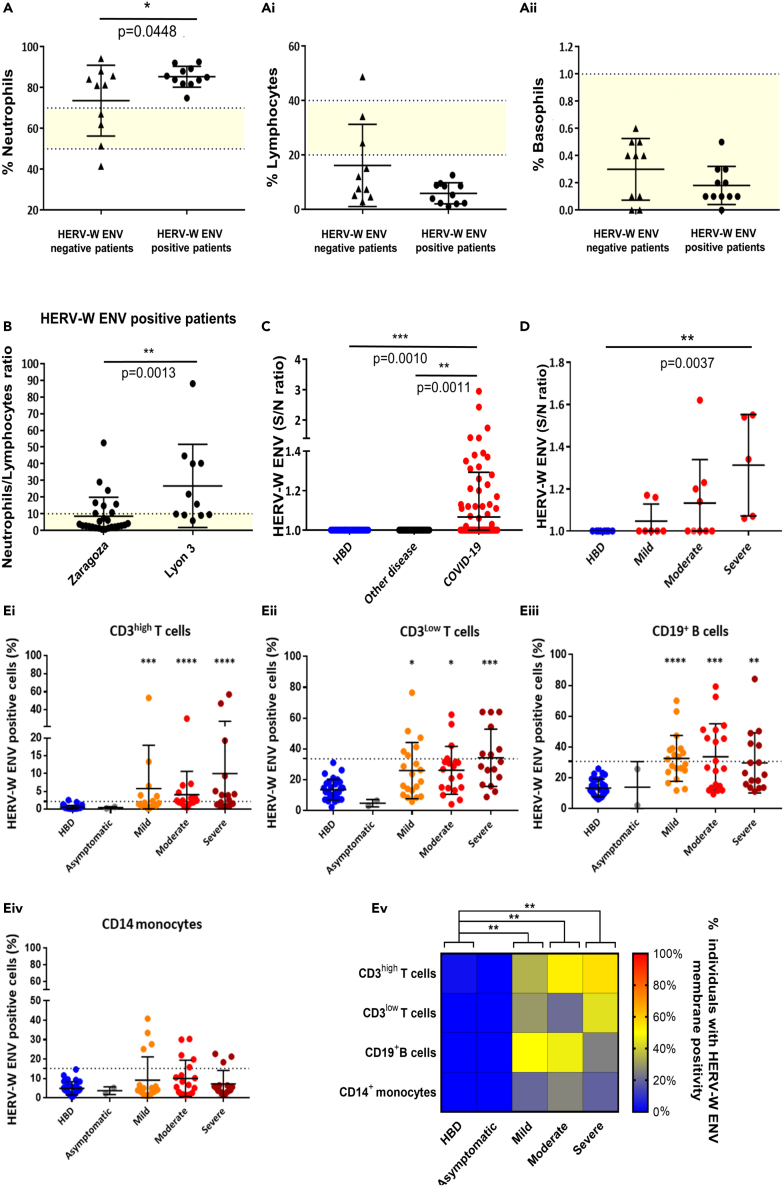
Prevalence of soluble HERV-W ENV in serum from COVID-19 patients correlates with blood biomarkers and disease severity (A) Scatterplots comparing COVID-19 patients either positive for HERV-W ENV in plasma (11 patients) or 10 negative patients with their corresponding percentage of polymorphonuclear neutrophils (Ai), lymphocytes (Aii), and polymorphonuclear basophils (Aiii) in the blood. Yellow areas represent the reference range for blood tests in healthy adults (QC-validated normal values provided with results). (B) Comparison of neutrophil/lymphocyte ratio between patients positive for HERV-W ENV from two different COVID-19 cohorts, early and late consisting in (i) a cohort of early post-PCR COVID-19 diagnosed outpatients from Zaragoza and in (ii) in patients (hospitalized) from “Lyon” cohort (cf. clinical data of Zaragoza and Lyon cohorts; https://doi.org/10.17632/3v4hfxv4w8.1) with symptomatic COVID-19 evolution. The normal interval for the neutrophil/lymphocyte ratio is represented by the colored area above the X axis. HBD: healthy blood donor. (C) Detection of soluble HERV-W ENV in COVID-19 patients’ serum at the day of RT-qPCR diagnosis (Zaragoza cohort, https://doi.org/10.17632/3v4hfxv4w8.1). HERV-W ENV detection in HBD (blue dots, n = 44), in non-COVID-19 patients (“other disease”, black dots, n = 43) and in COVID-19 patients (red dots, n = 143). (D) HERV-W ENV quantification in serum of HBD and COVID-19 patients presenting mild, moderate, or severe symptoms of the disease. (E) Results of cytofluorometry analyses on PBMC from “Lyon” cohort (https://doi.org/10.17632/3v4hfxv4w8.1) for each cell sub-populations CD3^high^ (Ei), CD3^Low^ (Eii), CD19^+^, (Eiii) and CD14^+^ (Eiv) according to the severity of the disease, respectively, in Ei to Eiv. Dotted lines correspond to the positivity threshold calculated from the mean values of HBDs ±3 SD. The Heatmap was generated by associating the severity of disease observed in COVID-19 patients with their profile based on the percentage of HERV-W ENV-positive patients (presented in color scale) for each cell subtype. It highlights the modification of HERV-W ENV profile detected at the PBMC surface depending on the symptom severity, with a shift of predominant HERV-W ENV^+^ T cells from mild to severe cases, and the opposite trend for HERV-W ENV^+^ B cells (Ev). Statistical analysis was performed as described in STAR Methods (∗p < 0.05, ∗∗p < 0.01, ∗∗∗p < 0.001 and ∗∗∗∗p < 0.0001).

Analyses of results from available blood analyses from hospitalized patients (“Lyon” cohort, patients #28 to #48) revealed a significant difference between polymorphonuclear neutrophils counts among patients with positive HERV-W ENV detection in serum and those among HERV-W-negative COVID-19 patients (Figure 4A). Interestingly, all neutrophil counts in HERV-W-positive patients (11/11) were above the physiological level. With an opposite distribution below the normal range, HERV-W-positive patients all had lymphopenia (Figure 4A). No difference was observed for other white blood cells, as exemplified with basophils (Figure 4A).

Furthermore, the ratio between neutrophils and lymphocytes counts (N/L), previously suggested to be a biomarker of COVID-19 severity,^5^ was significantly increased in more symptomatic COVID-19 cases positive for HERV-W ENV. This is illustrated with serum samples obtained from hospitalized patients (see clinical data of patients #28 to #48 from “Lyon” cohort, https://doi.org/10.17632/3v4hfxv4w8.1) with more and longer evolution, when compared with the cohort of early-diagnosed outpatients (symptomatic patients #49 to #191 from “Zaragoza” cohort, p = 0.0013) (Figure 4B). These results also highlight the difference in the disease clinical status of patients between the two cohorts, with early diagnosed versus cases later hospitalized.

Thus, to study the production of HERV-W ENV patients at early COVID-19 diagnosis, compared to patients with other diseases and healthy controls, the soluble HERV-W was quantified in sera from a cohort consisting of two successive sampling series of SARS-CoV-2 PCR-positive cases with heterogeneous clinical presentation (patients #49 to #191 from “Zaragoza” cohort, described in https://doi.org/10.17632/3v4hfxv4w8.1). A significant difference was still found between these COVID-19 patients compared to HBD or to other diseases (Figure 4C). Among these 143 early SARS-CoV-2 PCR-positive cases, 21% were positive for HERV-W ENV, unlike the 44 HBD, all negative for HERV-W. HERV-W ENV antigen was not detected in 43 sera from non-COVID-19 other diseases either.

Finally, patients were classified into mild, moderate, and severe groups using the clinical scale for COVID-19, recommended by the National Institute of Health of the United States (USA) guidelines (https://www.covid19treatmentguidelines.nih.gov/overview/clinical-spectrum/). The mean titers of HERV-W ENV in plasma (patients #28 to #48 from “Lyon” cohort) progressively increased with disease severity, and HERV-W ENV was detected in all cases with severe forms (Figure 4D). The comparison between the “severe” group and healthy controls was statistically significant despite low numbers of analyzed patients in this group. The analysis of cytofluorometry data obtained from patients classified using the same clinical scale is presented in Figure 4E (patients #1 to #27 and #192 to #221 from “Lyon” cohort, described in https://doi.org/10.17632/3v4hfxv4w8.1). The same strongly significant HERV-W ENV expression is observed in CD3^high^ T cells from mild cases but also increased from mild to severe cases in CD3^low^ T cells. HERV-W ENV detection was significant in CD19^+^ B cells but decreased from mild to severe cases. Comparison of CD-19^+^/HERV-W ENV^+^ B cell percentage increased with series of patients from Wuhan to Delta and Omicron variants (Figure 3B) and with decreasing severity of COVID-19, suggesting that HERV-W ENV^+^ B cells may be depleted with increasing disease severity. Finally, despite several cases with positive CD14^+^ cells, no statistical difference with asymptomatic cases or healthy controls was observed. The global evolution of the percentage of patients with HERV-W ENV^+^ PBMC sub-populations according to COVID-19 severity scale compared to controls is summarized in Figure 4E.

### Expression of SARS-CoV-2 and HERV-W ENV in tissues from autopsies of acute COVID-19 patients

**Figure 5:**
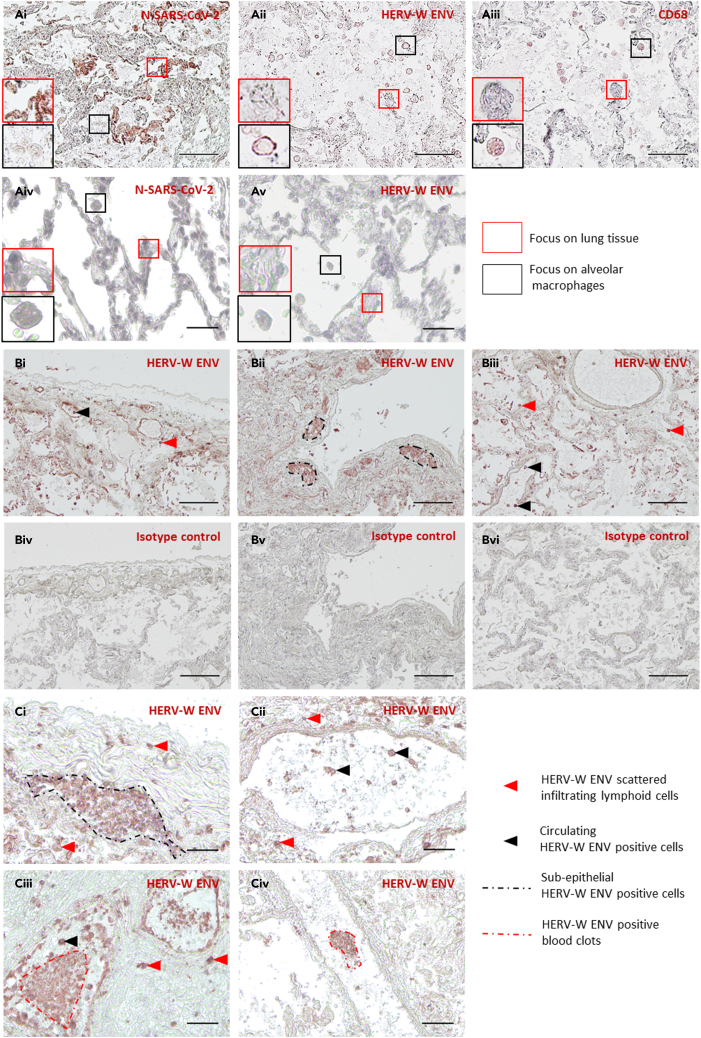
Immunohistology detection of HERV-W ENV and SARS-CoV-2 antigens in postmortem lung tissue from acute COVID-19 patients (A) N-SARS-CoV-2 (Ai, Aiv) and HERV-W ENV (Aii, Av) (brown-red staining) were immunodetected in lung tissue sections from necropsies of COVID-19 postmortem patients (Ai-Aiii) but not in non-COVID-19 (Aiv and Av), with representative examples of lung tissue from non-COVID-19 patients, comprising normal tissue surrounding lung tumors (presented: non-COVID-19 lung necropsy donor *D1*). To further confirm the phenotype of HERV-W ENV-producing macrophage-like cells seen in lung alveoli, CD68 immunostaining targeting a specific marker of macrophages was performed on sections from COVID-19 lungs (Aiii). Higher magnifications of pulmonary cells (Ai-Av, red squares) and of alveolar macrophages (Ai-Av, black squares) are presented on COVID-19 and non-COVID-19 tissues for comparison. (B) The specificity of HERV-W ENV immunodetection was also assessed on COVID-19 tissues by comparing anti-HERV-W ENV murine IgG1 GN_mAb_Env01 (BiBiii) and murine IgG1 isotype control (Biv-Bvi) at the same concentration (10 μg/mL) on adjacent tissue sections. (C) In addition to macrophage cells, specific HERV-W ENV staining on COVID-19 lung sections showed various HERV-W ENV-positive cell types, such as small round-shaped infiltrated cells with lymphoid morphology (Ci), isolated cells within large blood vessel lumen (Cii), blood vessel endothelium cells (Ciii), or multiple aggregated cells forming clots in blood vessels (Civ). Red arrowhead: HERV-W ENV scattered infiltrating cells; black arrowhead: circulating HERV-W ENV-positive cells; black dotted line: sub-epithelial infiltrated HERV-W ENV-positive cells; red dotted line: HERV-W ENV-positive blood clots. Ai-Av and Ci-Civ: Bars = 100 μm; BiBvi: Bars = 250 μm.

**Figure 6:**
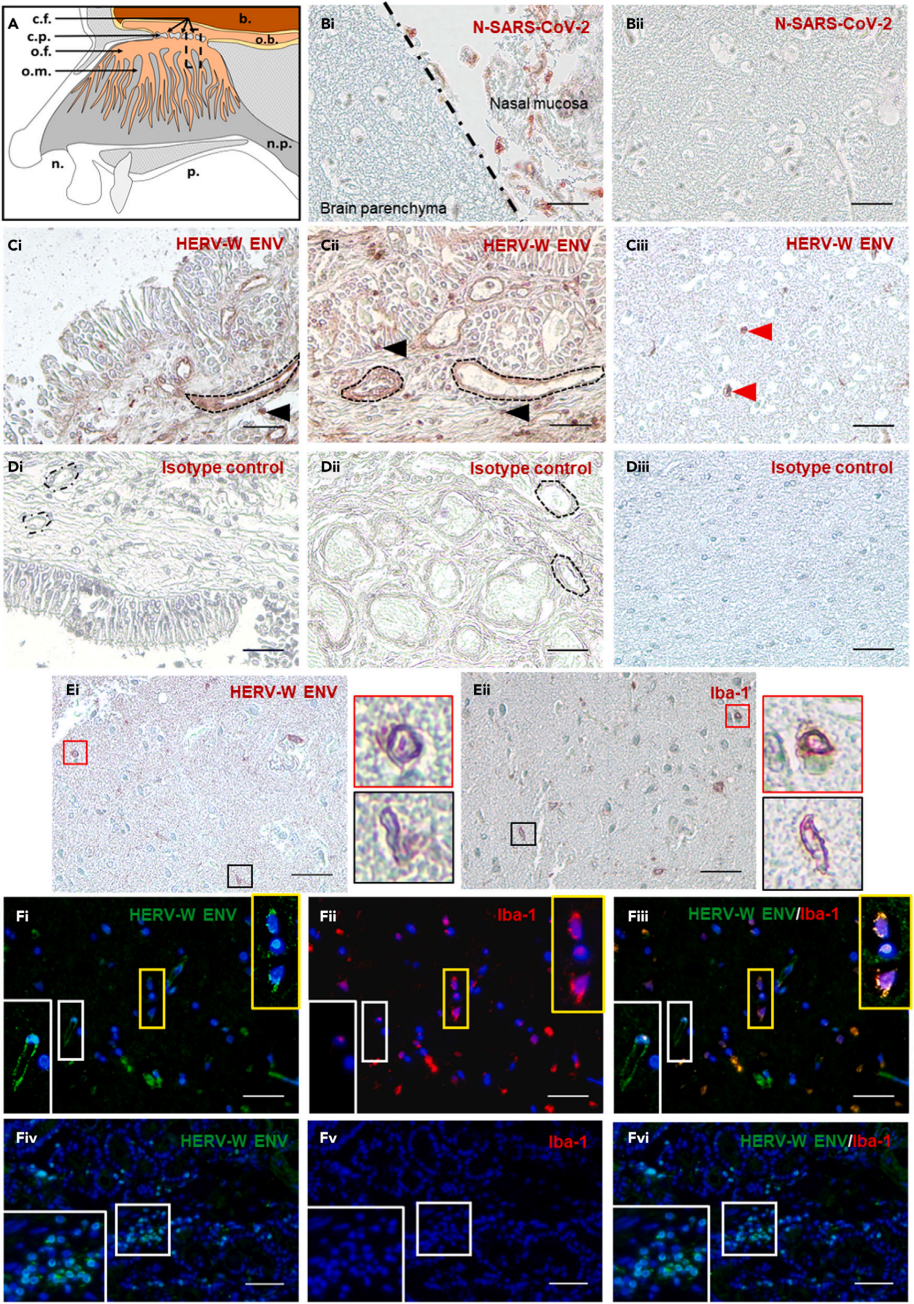
Immunohistology detection of SARS-CoV-2 and HERV-W ENV in postmortem brain parenchyma of the olfactory bulb and in nasal mucosa tissues from acute COVID-19 patients (A) Schematic representation of nasal cavity and location of tissue sampling. b.: brain; o.b.: olfactory bulb; n.p.: naso-pharynx; p.: palate; n.: nostrils; o.m.: olfactory mucosa; o.f.: olfactory fibers; c.p.: cribriform plate (ethmoid bone); cf.: cribriform foramina. (B) SARS-CoV-2 virus was searched for in brain parenchyma (Bi and Bii) and nasal mucosa (Bi) using anti-N SARS-CoV-2 immunodetection (brown-red staining). The black dotted line on Bi represents the boundary between brain parenchyma and nasal mucosa (position of the cribriform plate). SARS-CoV-2 nucleocapsid immunostaining was only observed in nasal mucosa and not in neighboring brain parenchyma (Bi). Bars = 100 μm. (C) HERV-W ENV immunodetection using the murine IgG1 antibody GN_mAb_Env01 (brown-red staining) revealed that HERV-W ENV IHC staining was detected in nasal epithelium (Ci), nasal mucosa (Cii), and brain parenchyma (Ciii). Bars = 100 μm. (D) Murine IgG1 isotype control (Di-Diii) was used at the same concentration (10 μg/mL) than anti-HERV-W ENV antibody without unspecfic background. Bars = 100 μm. (E) The detection of HERV-W in microglial cells was assessed using anti-HERV-W ENV (Ei) and anti-microglia-specific marker Iba-1 (Eii) in adjacent sections of brain parenchyma. The morphology of HERV-W ENV-positive cells is highlighted with higher magnification of small round cells (Ei, red square) and small elongated cells (Ei, black square). Higher magnifications of Iba-1-positive small round (Eii, red square) and small elongated cells (Eii, black square) are also boxed. Bars = 100 μm. (F) To further ascertain the production of HERV-W ENV by microglia, a double IF staining was performed on olfactory brain parenchyma and nasal mucosa: HERV-W ENV (Fi and Fiv, green label) and Iba-1 (Fii and Fv, red label). (Fiii) HERV-W ENV/Iba-1 double-positive cells (higher magnification: Fi-Fiii, yellow square). HERV-W ENV-positive/Iba-1-negative cells in brain parenchyma had the morphology of microvessel endothelial walls (higher magnification: Fi-Fiii, white square) and of lymphoid infiltrates in nasal mucosa (higher magnification: Giv-Gvi, white square). DAPI was used to stain nuclei (Fi-Fvi blue staining). Black dotted line: endothelial layer border; black arrowhead: HERV-W ENV-positive cells scattered in nasal mucosa; red arrowhead: HERV-W ENV-positive cells scattered in brain parenchyma. Bars = 100 μm.

To better understand the extent of HERV-W ENV expression in SARS-CoV-2-infected patients, we next analyzed the expression of this protein in tissues from COVID-19 patients (Figures 5, 6, and S4). Postmortem tissue samples from lung, heart, gastrointestinal tract, nasal mucosa, and brain were obtained from patients deceased from severe acute forms of COVID-19 (clinical data are provided in https://doi.org/10.17632/hmz5sm67rb.1).

Following the absence of HERV-K ENV detection in plasma of COVID-19 patients (Figure S3), despite positivity for HERV-W ENV (Figure 4C), we performed immunohistochemistry (IHC) staining with an already validated anti-HERV-K ENV monoclonal antibody targeting a highly conserved epitope.^15^^,^^35^ In accordance with results obtained with plasma, all tissue samples from all autopsied cases were negative for HERV-K ENV (Figure S3B).

Unlike the absence of the detection of HERV-K ENV, the lung tissue was readily stained for HERV-W ENV as well as SARS-CoV-2 N, although in different cell types (Figure 5). SARS-CoV-2 antigen was detected in lung epithelial cells but not in alveolar macrophages (Figure 5A). Conversely, HERV-W ENV antigen was strongly expressed at the cell membrane of CD68^+^ macrophages but not in neighboring SARS-CoV-2-positive epithelial cells (Figures 5B–5A). Furthermore, neither HERV-W ENV nor SARS-CoV-2 N staining was found within similar sections of lung tissue from non-tumoral regions of non-COVID patients with lung cancer, confirming the absence of non-specific staining (Figure 5A). In wider areas of COVID-19 lung tissue, HERV-W ENV expression was observed in scattered infiltrating lymphoid cells (Figure 5B) and with a sub-epithelial strong staining of cellular aggregates (Figure 5B), in parallel with the absence of staining with an isotype control in the same tissue samples (Figure 5B). HERV-W ENV-positive cells are presented with higher magnification in Figure 5C, which shows massive infiltrates of lymphoid cells, some of them diffusing within alveola and endothelial cells with aggregated cells or clots within blood vessels. In addition, an isolated blood clot representing a circulating micro-thrombus with strong HERV-W ENV staining was observed (Figure 5C).

In COVID-19 heart tissue samples (Figures S4A–S4C), HERV-W ENV was mainly found in endothelial cells from numerous small blood vessels, within cardiac muscle (Figure S4A) and in the pericardial fatty tissue (Figure S4B). The endothelial nature of HERV-W ENV-positive cells was confirmed with CD31 staining in similar vessel structures from neighboring slides (Figures S4A and S4B), and no detectable staining was seen with an isotype control (Figure S4C), confirming the absence of non-specific labeling. Surprisingly, no SARS-CoV-2 antigen was detected in cardiac tissues from studied COVID-19 samples.

Tissues from the gastrointestinal tract presented areas of strong labeling with SARS-CoV-2 anti-nucleocapsid antigen, mostly in epithelial cells but also in the gastric antral mucosa, in and around intestine sub-mucosal glands as well as goblet ting lymphoid cells (Figure S4E) and immune cells from mucosa-associated lymphoid tissue (MALT; Figure S4E). Here again, HERV-W ENV-expressing cells were different from those infected with SARS-CoV-2. However, a faint HERV-W ENV staining was often seen at the apical top of intestine epithelial cells (Figure S4E).

To further address the expression of HERV-W ENV in the central nervous system (CNS) of COVID-19 patients, as previously observed in MS lesions,^19^ we analyzed sections from tissue samples taken across the cribriform plate, comprising areas of the olfactory bulb and of the nasal mucosa (Figure 6). This anatomical region was chosen since it is suggested to be a most probable route of coronavirus passage to the brainstem via olfactory nerve roots within nasal mucosa^40^ and since frequently reported anosmia in COVID-19 patients indicates pathological involvement of the olfactory bulb parenchyma.^41^^,^^42^ Sections from samples with the upper anatomical brain region, i.e., the frontal lobe, were also analyzed.

Immunohistology analysis revealed both SARS-CoV-2 and HERV-W ENV protein expression in nasal mucosa areas. In sections at the CNS-nasal tissue interface (Figure 6B), a strong SARS-CoV-2 N staining was observed, indicating viral replication in nasal mucosa but not in neighboring CNS areas of the olfactory bulb. SARS-CoV-2 antigen was detected neither in various areas of CNS sections within the olfactory bulb nor in the frontal brain parenchyma (Figure 6B). A strong HERV-W ENV staining was seen in nasal mucosa, involving infiltrated lymphoid cells and, as already seen in pulmonary and cardiac tissues, the endothelium of blood vessels (Figure 6C). Conversely to SARS-CoV-2 antigen, HERV-ENV was detected both in the olfactory bulb and in the brain parenchyma within cells presenting the morphology of microglia (Figure 6E). Cells with the same morphology were stained by a microglial maker, Iba-1 (Figure 6E). Higher magnifications of HERV-W ENV-positive microglial cells within brain parenchyma are boxed and presented in Figure 6E, and the specificity of this staining was confirmed with isotype controls (Figure 6D). To further confirm the expression of HERV-W ENV in microglia within COVID-19 brain parenchyma, a double immunostaining was performed with HERV-W ENV and Iba-1-specific antibodies. Microglia-like cells positive for HERV-W ENV were co-stained with the antibody against Iba-1, thereby confirmed to be microglial cells (Figure 6F, boxed in yellow with higher magnification on the right side). On the same pictures, endothelial cells from an HERV-W ENV-positive blood vessel wall were consistently negative for Iba-1 (Figure 6F). In parallel, sections from nasal mucosa showed HERV-W ENV staining of infiltrated lymphoid cells, also negative for Iba-1 (Figure 6F).

**Figure 7:**
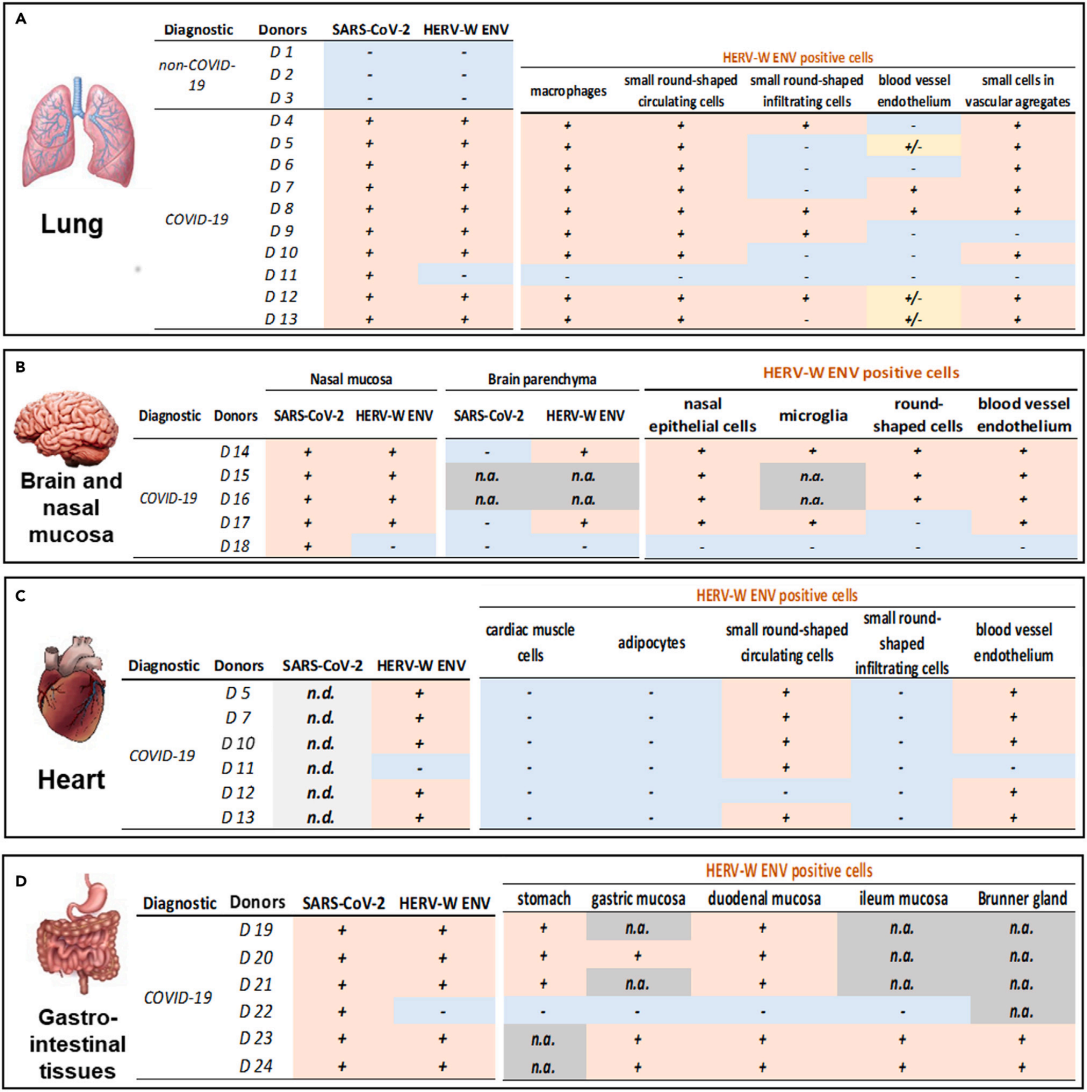
Summary of immunohistology detection of HERV-W ENV and N-SARS-CoV-2 proteins in all studied tissues from COVID-19 patients (A and B) Detection of HERV-W ENV and SARS-CoV-2 N is presented for each donor, for all analyzed organs. (A) lungs (B) brain and nasal mucosa/epithelium. (C and D) (C) heart and (D) digestive tract. Donors (D) 1–3 of lung tissue are obtained from non-covid patients from CRB Lyon, while donors D 4–24 are from postmortem COVID-19 patients from Mexico City hospital. Representative immunohistology results are presented in Figures 5, 6, and S4 (n.d. not detected; n.a. not available). The analysis of potential tissue cross-reactivity in healthy human organs, obtained in similar postmortem conditions and including the presently studied tissues, showed no significant staining and, thereby, no expression of HERV-W ENV protein (described in STAR Methods). Isotype controls have been used on all tissues, which constantly resulted with negative staining, and the negative results in staining obtained with anti-HERV-K monoclonal also provided another “isotype control” in the corresponding Figures.

Immunohistochemical results for HERV-W ENV and SARS-CoV-2 N protein staining from studied tissues of COVID-19 autopsies are summarized in Figure 7. Altogether, these results revealed that HERV-W ENV is expressed in postmortem tissues of lungs, gut, heart, brain parenchyma, and nasal mucosa from acute COVID-19 patients in cell types relevant for COVID-19-associated pathogenesis within affected organs.

## Discussion

Despite rapid advances in basic and translational science, COVID-19 continues to pose an important global health threat. This new disease revealed heterogeneous clinical profiles in the evolution of acute and post-acute COVID-19, with symptoms not directly related to the viral infection. In the present study we have analyzed the induction of HERV expression during COVID-19 disease. Our initial *in vitro* results showed that SARS-CoV-2 can trigger both HERV-W and HERV-K *ENV* RNA transcription after a single exposure to SARS-CoV-2 virus, but only HERV-W ENV protein expression in short-term PBMC cultures from about 30% of healthy donors. These divergent outcomes suggest heterogeneity in the healthy population for SARS-CoV-2-induced HERV activation.

Recombinant wild-type SARS-CoV-2 spike protein trimer induced the production of IL-6 as previously reported^43^ in PBMC from all donors either responding or not with HERV activation, at time points posterior to the activation of HERVs. Thus, a cytokine expression such as IL-6 secretion is not likely to be responsible for the induction of HERV-W ENV expression. Cytofluorometry analysis confirmed that HERV-W ENV protein *in vitro* early expression was predominantly and strongly induced in CD3^low^ T lymphocytes within the CD3^+^ T cell population. Consistently, T lymphocytes that underwent a very recent contact with antigenic components of SARS-CoV-2 may dynamically become activated CD3^+^ T cells as previously described.^37^ Alternatively, in COVID-19 patients, our observations may corroborate previous reports describing superantigen motifs of SARS-CoV-2 spike protein,^44^ which may involve cellular mechanisms associated with lymphopenia and hyperinflammation as already characterized for other emerging viruses (e.g., Ebola or Lassa).^45^^,^^46^ However, because HERV-W ENV has also been shown to display superantigen-like effect,^22^ the origin of this short-term effect on T lymphocytes may be questioned. Results from a recent study potentially provide an answer, since showing correlated expression between HERV-W ENV and markers of exhaustion in T lymphocytes from severe COVID-19 cases.^27^ Altogether, results showed (i) lymphopenia in all HERV-W ENV-positive COVID-19 hospitalized cases from the same cohort along with (ii) a significant difference for the lymphocyte/neutrophils ratio between cohorts of early-diagnosed patients, asymptomatic or with mild disease, versus hospitalized patients in later stages of COVID-19. These observations suggest a negative impact of this endogenous protein on the fate of expressing lymphocytes, as previously suggested by the co-labeling of HERV-W ENV with exhaustion markers on T cells from COVID-19 patients.^27^

HERV activation occurs without signs of infection of lymphocytes by SARS-CoV-2. Consistently, a recombinant trimer of its wild-type spike protein without stabilizing mutations^39^ appeared sufficient to reproduce similar HERV-W and -K *ENV* RNA stimulation as well as HERV-W ENV protein production in lymphoid cells, albeit in a subset of donors like with the infectious virus. This type of HERV-W activation mediated by an interaction between a triggering virus and a specific receptor on certain cells has already been described, e.g., CD46 receptor with HHV-6A.^38^ As SARS-CoV-2 induces HERV-W ENV expression in human lymphoid cells that do not express ACE2, yet undetermined receptor(s) are expected to mediate HERV activation by SARS-CoV-2, which should now be further studied with recent data on alternative receptors.^47^

In addition to the SARS-CoV-2-induced activation of HERV-W expression in cultured lymphocytes, this study has demonstrated their activation in COVID-19 patients. In hospitalized individuals with different severity status, HERV-W ENV protein was confirmed to be expressed at the membrane of significant proportion of both CD3^low^ and CD3^high^ T cells. A significant expression was also found in B lymphocytes, which corresponds to a cell type already shown to be permissive for HERV-W expression.^48^^,^^49^ Interestingly, HERV-W ENV^+^/CD19^+^ B lymphocytes were most prevalent and represented the most significant percentage in PBMCs from patients infected with more recent SARS-CoV-2 variants, Delta and Omicron, which might be related to particular properties of this variants. This would suggest a more rapid detrimental effect on HERV-W ENV-expressing B versus T cells, thereby possibly depleting positive B lymphocytes in later and more severe disease evolution. Of note, HERV-W expression was not previously observed in T cells in any pathological conditions, before its first observation in COVID-19,^27^ suggesting the specific capacity of SARS-CoV-2 spike to trigger its expression in T lymphocytes, possibly followed by a longer survival in the bloodstream than B lymphocytes or monocytes, which were seen as strongly expressing macrophages after *in vitro* induction in the present study. Furthermore, analyses of other blood parameters from studied patients showed that their neutrophil counts, reported to be most often increased in COVID-19 with worsening evolution,^50^^,^^51^ also paralleled HERV-W ENV presence in the serum, with values above the upper normal limit in all hospitalized patients with ENV-positive plasma.

After the recent characterization of a soluble hexameric form of HERV-W ENV in MS brain lesions,^15^ the present study has shown its presence in the form of a circulating protein in COVID-19 plasma or sera. In a series from patients in intensive care unit, a significant correlation was found between HERV-W ENV detection on CD3^low^ and CD3^high^ T lymphocytes and the soluble protein in plasma. Though moderate, this nonetheless statistically significant correlation was seen between blood cells and the corresponding plasma devoid of cells, which should indicate a relationship between HERV-W-expressing lymphocytes and the release of this soluble protein in blood. The presence of HERV-W ENV in plasma was confirmed to be significantly increased in hospitalized patients with severe COVID-19 but was not detected in healthy controls, whereas HERV-K ENV protein was never detected in plasma or serum. Since all severe COVID-19 cases were tested positive for HERV-W ENV protein in plasma, this points to a potential marker of disease severity as already shown with blood T lymphocytes.^27^ We also found HERV-W ENV in plasma of about 20% of early COVID-19 cases who were included after positive SARS-CoV-2 PCR result, independently of disease severity. This percentage is similar to that of healthy donors who showed HERV-W ENV positivity in PBMC exposed to SARS-CoV-2 challenge *in vitro*. Altogether, these data suggest that a percentage of individuals with an underlying susceptibility to more symptomatic and/or severe evolution may be linked to the activation of HERV-W ENV expression. In PBMC cultures non-responding to SARS-CoV-2 exposure, we had observed HERV RNA levels below the levels of non-exposed controls, which may be explained by the activation of HERV-inhibitory pathways and effectors. Thus, an inter-individual variability in the potency of HERV-inhibitory mechanisms, possibly with an (epi)genetic origin, may provide an explanation for non-universal activation of HERV-W ENV upon SARS-CoV-2 challenge, similarly to individuals with COVID-19. Indeed, most SARS-CoV-2 RT-PCR-positive individuals do not develop major symptomatology after infection, including asymptomatic cases who may represent about 35% of PCR-positive cases.^52^ Moreover, since patients from different geographic areas and time periods of the pandemic, infected by different variants of SARS-CoV-2 were analyzed, the global results of this study are not expected to be influenced by such variables.

The analysis of tissues obtained at autopsy from patients deceased from acute COVID-19 revealed that HERV-W ENV was strongly expressed in numerous tissues, including lymphoid infiltrates surrounding lung alveola, within nasal or intestinal mucosa, and in endothelial cells from blood vessels of all tissues including the CNS parenchyma. In addition, HERV-W ENV expression in the CNS was found within scattered cells that were confirmed to be microglia. Strong HERV-W ENV staining was also often detected in aggregated cells corresponding to thrombotic structures in blood vessels of lung samples. SARS-CoV-2 nucleocapsid, corroborating viral replication, was readily detected in epithelial cells within lungs or intestines, as well as nasal mucosa, but not in analyzed sections from brain parenchyma, nor in the cardiac tissues of the studied cases. It was not even detected in the CNS parenchyma from olfactory bulb sections neighboring nasal mucosa with noticeable ongoing SARS-CoV-2 infection. Finally, a very strong expression of SARS-CoV-2 was observed in intestinal epithelium coinciding with numerous HERV-W ENV-positive infiltrated lymphoid cells in the mucosa. HERV-W ENV expression was also found in intestine, in lymphoid tissue (MALT) next to SARS-CoV-2-positive areas. Beyond this detection in the gastrointestinal tract of acute COVID-19 cases, it should be relevant to mention that persisting SARS-CoV-2 infection in the digestive system has been reported.^53^ Of note, the specificity of anti-HERV-W ENV antibody used in this study and the absence of significant cross-reactivity on postmortem healthy human tissues from all organs as well as the absence of any pharmacokinetic or toxic effect related to elevated doses in clinical trials with healthy volunteers have been documented and reported previously.^54^^,^^55^ This was more specifically further studied for brain, blood vessels, and pancreas tissues with same results.^18^^,^^19^^,^^24^^,^^28^^,^^56^^,^^57^ In addition, HERV-K ENV has not been detected in the human sera and tissues from healthy donors when using the same antibody as used in this study; nevertheless, the same antibody was previously shown to efficiently detect HERV-K ENV in the cerebrospinal fluid of patients with amyotrophic lateral sclerosis.^35^

Globally, cells expressing HERV-W ENV did not correspond to cell phenotypes seen to be infected with SARS-CoV-2, consistently with our observations of HERV-W ENV protein expression in lymphocytes without infection by SARS-CoV-2. Most importantly, data presented here show HERV-W ENV expression in tissue-infiltrated lymphoid cells within affected organs, similarly to what is observed in blood of COVID-19 patients. Therefore, results from IHC analyses indicate that HERV-W ENV expression is intimately associated with organs and cells involved in COVID-19-associated or superimposed pathology, e.g., in vasculitis or intravascular thrombotic processes.^8^^,^^57^ Moreover, given the known HERV-W involvement in the microglia-driven pathogenesis of MS^18^^,^^19^^,^^24^ or of certain psychoses associated with inflammatory biomarkers,^58^^,^^59^ the presently observed HERV-W ENV expression in microglia strongly suggests a role in neurological symptoms and cognitive impairment mostly occurring or persisting during the post-acute COVID-19 period.^41^^,^^42^^,^^60^^,^^61^^,^^62^

In acute primary infection, the pathogenic effects of HERV-W ENV on immune cells need to be further considered to better understand COVID-19 immunopathogenesis. HERV-W ENV production may result in a hyperactivation of the innate immunity via HERV-W ENV-mediated Toll-like receptor 4 (TLR4) activation^63^ and in a possible contribution to the frequently observed lymphopenia along with an adaptive immune defect. The induction of autoimmune manifestations^64^^,^^65^^,^^66^^,^^67^ as previously shown to be provoked with HERV-W ENV, initially called multiple sclerosis associated retrovirus (MSRV) envelope protein,^21^ as well as the capacity of HERVs to modulate innate immunity should also be considered.^68^

Altogether, these data indicate that HERV-W ENV does not simply represent a biomarker of COVID-19 severity or evolution but is also likely to be a superimposed pathogenic player contributing to the disease severity and may help to explain the inter-individual variability in COVID-19 manifestations. In addition, it may play a role in the clinical evolution with possible long-term pathology as seen with the now-emerging post-COVID secondary pandemic^60^^,^^69^ representing millions of patients suffering from various symptoms and long-term disabling pathology for which no rationalized understanding or therapeutic perspective can be proposed to date. In face of this challenging situation, data from the present study strongly suggest HERV-W ENV as a marker of severity and as a potential therapeutic target for personalized medical approaches in COVID-19-associated syndromes.

### Limitations of the study

Further understanding the regulation of the expression of HERV-W and the signaling pathway used by SARS-CoV-2 to induce it will be important in the future work, to better apprehend the implicated mechanisms. In addition, the exploration of the effect of HERV-W ENV on the hyperactivation of innate immunity seen in COVID-19 patients and further in-depth studies of its role in the fate of T lymphocytes should help in explaining the role of HERV-W in the immunopathogenesis of certain COVID-19-associated syndromes.

## STAR★Methods

### Key resources table

**Table:** 

REAGENT or RESOURCE	SOURCE	IDENTIFIER
**Antibodies**		

Anti-HERV-W ENV (coupled or not with biotin or FITC)	Geneuro (in house)	GN_mAb_Env01
Anti-HERV-K ENV	Geneuro (in house)	GN_mAb_K01
Mouse IgG1 isotype	R&D Biotech	MAB002 ; RRID:AB_357344
Mouse IgG1 isotype-FITC	Miltenyi Biotec	130-113-199 ; RRID:AB_2733683
Anti-CD3-PE	BD Biosciences	552127 ; RRID:AB_394342
Anti-CD14-PerCP	BioLegend	301848 ; RRID:AB_2564059
Anti-CD14-BV421	BD Biosciences	563743 ; RRID:AB_2744289
Anti-CD19-APC-H7	BD Biosciences	560252 ; RRID:AB_1645468
Anti-CD19-APC-Cy7	BD Biosciences	557791 ; RRID:AB_396873
Anti-CD68	Abcam	ab955 ; RRID:AB_307338
Anti-CD31	Abcam	ab28364 ; RRID:AB_726362
Anti-Iba1	Wako	019–19741 ; RRID:AB_839504
Anti-N-SARS-CoV-2	NOVUS	NB100-56576 ; RRID:AB_838838
Anti-N-SARS-CoV-2	SinoBiological	40143-T62
Anti-*S*-SARS-CoV-2	SinoBiological	40590-T62
Goat anti-Mouse-HRP	Abcam	ab6789 ; RRID:AB_955439
Goat anti-Mouse-Alexa488	ThermoFisher	A21202
Goat anti-Rabbit-HRP	Abcam	ab6721 ; RRID:AB_955447
Goat anti-Rabbit-Alexa555	ThermoFisher	A32732

**Bacterial and virus strains**		

SARS-CoV-2	BetaCoV/France/IDF057½020	GISAID Accession ID: EPI_ISL_411218

**Biological samples**		

Healthy blood donor’s PBMCs, sera and plasma	“Etablissement Français du Sang” of Lyon (France)	
“Lyon cohort” COVID-19 donor’s PBMCs, sera and plasma	biobank of “Hospices Civils de Lyon” (France)	
“Zaragoza cohort” COVID-19 and healthy blood donor’s PBMCs, sera and plasma	“Biobanco del Sistema de Salud de Aragon” (Spain)	
Non-COVID-19 paraffin tissue slides	biobank of “Hospices Civils de Lyon” (France)	
COVID-19 paraffin tissue slides	National Institute of Respiratory Diseases in Mexico City (Mexico)	

**Chemicals, peptides, and recombinant proteins**		

non-stabilized trimer Spike SARS-CoV-2	ACROBiosystems	SPN-C52H8
DAPI	Sigma	D9542
LPS-EK	Invitrogen	Tlrl-eklps
Polymyxin B	Invitrogen	Tlrl-pmb
Fos Cholin 16	Anatrace	F316S-1 GM
Protease Inhibitor Cocktail	Roche	5892791001

**Critical commercial assays**		

Wes	ProteinSimple	Wes
The SARS-CoV-2 Multi-Antigen Serology Module	ProteinSimple	SA-001
Mouse detection module for Simple Western	ProteinSimple	DM-002
Opt EIA Set Human IL-6	BD	555 220
CellTiter-Glo 2.0 Assay kit	Promega	G9241
Protein Deglycosylation kit – denaturing buffer	NEB	B6045S
Protein Deglycosylation kit – enzymes mix	NEB	P6044S
StepOnePlus – Real-Time PCR System	Applied Biosystems	4376600

**Deposited data**		

Results related to Figure 1A - Healthy Blood Donors (HBD) https://data.mendeley.com/datasets/rc74sdgksk/1	Additional supplemental items are available from Mendeley Data	https://doi.org/10.17632/rc74sdgksk.1
Clinical data: COVID-19 patient cohorts from Lyon and Zaragoza https://data.mendeley.com/datasets/3v4hfxv4w8/1	Additional supplemental items are available from Mendeley Data	https://doi.org/10.17632/3v4hfxv4w8.1
Clinical data: Brain Necropsies - Mexico city collection https://data.mendeley.com/datasets/hmz5sm67rb/1	Additional supplemental items are available from Mendeley Data	https://doi.org/10.17632/hmz5sm67rb.1

**Experimental models: Cell lines**		

Monkey VERO E6 cell line	ATCC	CRL1586 ; RRID:CVCL_0574
Human Calu-3 cell line	ATCC	HTB-55

**Oligonucleotides**		

HERV-W *ENV* forward primer	In house	GTATGTCTGATGGGGGTGGAG
HERV-W *ENV* reverse primer	In house	CTAGTCCTTTGTAGGGGCTAGAG
HERV-K *ENV* forward primer	In house	CTGAGGCAATTGCAGGAGTT
HERV-K *ENV* reverse primer	In house	GCTGTCTCTTCGGAGCTGTT
*N*-SARS-CoV-2 forward primer	In house	AAACATTCCCACCAACAG
*N*-SARS-CoV-2 reverse primer	In house	CACTGCTCATGGATTGTT
*ACE2* forward primer	In house	TCCATTGGTCTTCTGTCACCCG
*ACE2* reverse primer	In house	AGACCATCCACCTCCACTTCTC
*B2M* forward primer	CellCarta	TTACTCACGTCATTCAGCAG
*B2M* reverse primer	CellCarta	GATGGATGAAACCCAGACAC
*GAPDH* forward primer	CellCarta	CACCCACTCCTCCACCTTTGAC
*GAPDH* reverse primer	CellCarta	AGACCATCCACCTCCACTTCTC

**Software and algorithms**		

Compass	ProteinSimple/Biotechne	RRID:SCR_022930
ImageJ	Schneider et al.^70^	https://imagej.nih.gov/ij/ ; RRID:SCR_003070
FlowJo	FlowJo	v.10 ; RRID:SCR_008520

**Other**		

Streptavidin-FITC	eBioscience	11-4317-87
Streptavidin-Alexa647	Invitrogen	S32357
Kit Cytofix/Cytoperm	BD	51-2090KZ
Perm/Wash buffer	BD	51-2091KZ
FcR Blocking Reagent, human	Miltenyi Biotec	130-059-901
3 wells epoxy microscope slides	Thermo Scientific	30-12A-BLACK-CE24
Fluoromount-G mounting medium	Southern Biotech	0100–01
NIKON Eclipse TSR2 microscope	NIKON	Eclipse TSR2
BD LSR Fortessa	BD	LSR Fortessa
Ultra-Comp eBeads plus Compensation beads	Invitrogen	01-3333-42
GloMax plate reader	Promega	GM3000 ; RRID:SCR_015575
AMICON Ultra-0.5 100K	Merk-Millipore	AMICON Ultra-0.5 100K

### Resource availability

#### Lead contact

Further information and requests for reagents and resources should be directed to and will be fulfilled by the lead contact.

#### Materials availability

Requests for new materials generated in this paper are to be directed to and will be fulfilled (pending MTA and associated restrictions) by the lead contact.

### Experimental model and subject details

#### Study design

The objective of this study was to analyze the implication of endogenous retroviruses (HERVs), and in particular the envelope protein of the HERV-W family (HERV-W ENV), in the pathophysiology of COVID-19. Certain exogenous viruses are known to be capable of awakening HERV sequences which may have synergistic pathogenic effect with the agent initially responsible for the infection. In order to analyze if that may be the case with SARS-CoV-2 infection, we opted for a two-step strategy: 1. Study in culture *in vitro* whether SARS-CoV-2 and more particularly the Spike protein is capable of inducing the expression of HERV-W ENV in PBMCs of healthy blood donors; 2. Analysis of samples from COVID-19 patients, including PBMCs, plasma, serum and post-mortem tissues of lungs, hear, gastrointestinal tract and nervous tissue. All experiments were reproduced at least three times when possible depending on the availability of the biological material studied. All graphical plots show the mean +standard deviation. All other imaging or flow cytometry data show a representative example of the indicated total number of experiments. For all the immunodetection, all the specificity controls were carried out (isotype control, positive control, negative control, evaluation of the background noise in the absence of primary antibody).

#### Ethical approval

All healthy blood donors signed a written Informed Consent Form, documented at the French Blood Center (EFS), allowing the use of their blood and blood components for medical research after anonymization. Provision of samples of “Lyon cohort” from the biobank of “Hospices Civils de Lyon” was approved by the ethical committee (Center de Ressources Biologiques des Hospices Civils de Lyon, Hôpital de la Croix-Rousse, Lyon France) and French Ministry of Research for the constitution of a collection of COVID-19 biological samples and their session for the purpose of research (Authorization N°: DC-2020-3919 and AC-2020-3918). The provision of samples of “Zaragoza cohort” from the “Biobanco del Sistema de Salud de Aragon” (PT20/00112) and the study protocol were approved by the scientific advisory board of the Biobank and by the local ethics committee (CEICA) (protocol C.P. - C.I. PI21/153 (07/04/21). The biobank is integrated into Spanish National Biobanks Network, Instituto de Salud Carlos III, Madrid, Spain. All samples and data from patients were processed following standard operating procedures. Paraffin embedded COVID-19 tissue slides (lung, heart, nasal and brain tissue) from 15 patient necropsies were provided by the National Institute of Respiratory Diseases in Mexico City, Mexico (Ethical committee approval and legal authorization: Autorización para realizer estudios post-mortem INER-SAM-01, Mayo 2021, Instituto Nacional de Enfermedades Respiratorias, Secretaría de Salud, México. Licencia Sanitaria No. 12-AM-09-012-0002. Protocolo B12-20: Identificación de los Factores Inmunológicos Relacionados con el Control de la Infección por SARS- CoV-2 years la Gravedad de la Enfermedad COVID-19).

#### Cells

Vero E6 and Calu-3 cells were grown in DMEM Glutamax (Thermo) supplemented with 10% fetal bovine serum (FBS), glutamine and antibiotics (100 U/mL of penicillin and 100 μg/mL of streptomycin) in 5% CO2 incubators at 37°C and were tested negative for mycoplasma spp. Peripheral blood mononuclear cells (PBMC) isolated by Ficoll separation (Ficoll-Plaque PLUS) (GE Healthcare, 17-1440-02) from blood samples and cultured in RPMI-1640 medium (Gibco, 61870-010) completed with 5% of decomplemented Human AB serum (Sigma, H4522). Healthy donors signed a written Informed Consent Form, documented at the EFS, allowing the commercial use of their blood and blood components for medical research after definite anonymization. 57 PBMC and corresponding plasma (Lyon cohort patients #1 to #27 and #192 to #221) and 20 sera (Lyon cohort patients #28 to #48) from SARS-CoV-2-positive individuals were obtained from the biobank of “Hospices Civils de Lyon” with anonymized clinical and biological data (https://doi.org/10.17632/3v4hfxv4w8.1). Upon receipt, PBMC were cultured 24 h in RPMI-1640 medium (Gibco, 61870-010) completed with 5% of decomplemented Human AB serum (Sigma, H4522). Sera of 44 healthy controls (unknown COVID-19 status; pre-pandemic sampling), 43 SARS-CoV-2 PCR-negative patients with other diseases and 143 COVID-19 patients (SARS-CoV-2 PCR positive) as well as anonymized. Clinical and biological data of the 143 COVID-19 patients were provided as “Zaragoza cohort” (patients #49 to #191) at https://doi.org/10.17632/3v4hfxv4w8.1. Samples and data from patients were processed following standard operating procedures.

#### Antibodies

Several primary antibodies were used for specific immunodetection using different approaches. For cytofluorometry analysis the biotinylated version of the anti-HERV-W ENV antibody (GN_mAb_Env01-biotin, Geneuro, used at 10 μg/mL) was used, followed with streptavidin-FITC or streptavidin-Alexa647 depending on the multi-immunolabeling strategy (11-4317-87, eBioscience and S32357, Invitrogen, respectively). This antibody is used in combination with directly labeled commercial antibodies: anti- CD3-PE (552127, BD Biosciences, diluted 1/5), anti-CD14-PerCP (301848, BioLegend, diluted 1/20), anti-CD14-BV421 (563743, BD Biosciences, diluted 1/20), anti-CD19-APC-H7 (560252, BD Biosciences, diluted 1/20) and anti-CD19-APC-Cy7 (557791, BD Biosciences, diluted 1/20). For simple Western blot analysis murine monoclonal anti-HERV-W ENV (GN_mAb_Env01, Geneuro, 20 μg/mL) and murine monoclonal anti-HERV-K ENV (GN_mAb_EnvK01, Geneuro, 30 μg/mL) were used and blots were revealed using a ready to use mouse detection module for simple western (DM-002, ProteinSimple). For immunofluorescence and immunohistology following mAbs were used: murine monoclonal anti-HERV-W ENV (GN_mAb_Env01, Geneuro, 10 μg/mL), mouse IgG1 isotype control (MAB002, R&D Biotech, 10 μg/mL), murine monoclonal anti-HERV-K ENV (GN_mAb_EnvK01, Geneuro, used for IHC at 10 μg/mL) the commercial antibodies anti-CD68 (murine, ab955, Abcam, diluted 1/3000 after antigen retrieval 10 min 95°C in citrate buffer pH9), anti-CD31 (rabbit, ab28364, Abcam, diluted 1/50 after antigen retrieval 20 min 95°C in citrate buffer Tris EDTA pH6), anti-N SARS-CoV-2 (rabbit, NB100-56576, NOVUS, diluted 1/50 after antigen retrieval 10 min 95°C in citrate buffer pH6) and anti- Iba1 (rabbit, 019–19741, Wako chemicals, diluted 1/500 after antigen retrieval 10 min 95°C in citrate buffer pH6). Binding of primary antibodies was revealed using coupled secondary antibodies: goat anti-mouse-HRP (ab6789, Abcam), goat anti-rabbit-HRP (ab6721, Abcam), goat anti-mouse-Alexa488 (A21127, ThermoFischer), goat anti-rabbit-Alexa555 (A32732, ThermoFischer), all used at dilution 1/1000. The specificity of anti-HERV-W ENV antibody has been validated in immunobiological studies, by the detection of its target protein in tissues and body fluids from patients with associated diseases and by the absence of HERV-W ENV detection or antibody cross-reactivity in healthy human tissues, including the brain, lung, heart and intestinal tissues, for pre-clinical regulatory analyses (ClinicalTrials.gov Identifier of human safety studies: NCT02452996, NCT03239860, NCT01699555, NCT01639300, NCT03574428). The specificity of this antibody and the absence of target protein expression in healthy humans has also been reported in several previous publications^15^^,^^18^^,^^19^^,^^24^^,^^28^^,^^55^^,^^71^ and the specificity of anti-HERV-K ENV antibody in samples from patients with Amyotrophic Lateral Sclerosis.^35^

### Method detail

#### Exposure to SARS-CoV-2 and to recombinant SARS-CoV-2 spike trimer

SARS-CoV-2 strain (BetaCoV/France/IDF0571/2020, GISAID Accession ID: EPI_ISL_411218) was cultured on Vero E6 cell line (ATCCCRL1586) for virus production. All experiments were performed with freshly prepared PBMC, collected on Ficoll from whole blood of healthy blood donors. PBMC and Vero cells were infected with SARS-CoV-2 at a multiplicity of infection (MOI) 0.1. PBMC inoculation with SARS-CoV-2 infectious virus at 0.1 MOI was performed in RPMI-1640 medium (Gibco, 61870-010) completed with 2% of heat inactivated Human AB serum (Sigma, H4522). Infection of Vero cells was performed in DMEM medium (DMEM, GibcoTM) completed with 2% of heat inactivated FCS. 2 h later, the concentrations of AB-human serum or FCS were increased to 10%. PBMC, 4x105 cells per well, were cultured in 48 wells plate in presence of RPMI 1640 (Gibco, 61870-010) completed with 5% of heat inactivated AB human serum (Sigma, H4522). Treatment with recombinant non-stabilized trimer Spike SARS-CoV-2 (ACROBiosystems, USA; SPN-C52H8, containing <1 Endodoxin Unit (EU)/μg of protein) was performed at 0.5 and 2.5 μg/mL.

#### Serology

SARS-CoV-2 serology of blood donors was determined in plasma diluted 10 times, using Simple Western technology, an automated capillary-based size sorting and immunolabeling system (ProteinSimpleTM). The SARS-CoV-2 Multi-Antigen Serology Module (SA-001) was used with Wes device and all procedures were performed according to manufacturer’s protocol. Wes device was associated with Compass software for device settings and raw data recording (ProteinSimple/Biotechne).

#### Immunofluorescence (IF)

Cells in suspension were pelleted by centrifugation and deposited on 3 well epoxy microscope slides (Thermo Scientific, 30-12A-BLACK-CE24) while adherent cells were manipulated directly in 48 wells plates. Suspension and adherent cells received the same following steps. Cells were fixed in paraformaldehyde 4% during 15 min at RT. Cells were washed tree times in 1X PBS and permeabilized 15 min in 0.2% Tween 20, 1X PBS. Saturation was performed using 2.5% horse serum, 0.2% Tween 20, 1 X PBS, during 30 min at room temperature before incubation with a mix of primary antibodies during 1 h or overnight: 3 μg/mL of anti-HERV-W ENV (GeNeuro, GN_mAb_Env01, murine antibody) or 10 μg/mL anti-HERV-K ENV (GeNeuro, GN_mAb_Env-K01, murine antibody), together with either anti-N SARS-CoV-2 diluted 1/500 (SinoBiological, 40143-T62, rabbit antibody) or anti-S SARS-CoV-2 diluted 1/500 (SinoBiological, 40590-T62, rabbit antibody). Antibody solutions were prepared in the previously described saturation buffer. After three washes in PBS 1X, cells were incubated during 1 h with secondary antibodies mix containing 1 μg/mL goat anti-mouse Alexa Fluor 488 (ThermoFisher Scientific, A11029), 1 μg/mL donkey anti-rabbit Alexa Fluor 647 (ThermoFisher Scientific, A31573) and DAPI ½000 (Sigma, D9542) diluted in the previously described saturation buffer. Finally, cells were washed three times in 1X PBS and mounted using the Fluoromount-G mounting medium (Southern Biotech, 0100-01). Images were acquired on NIKON Eclipse TS2R microscope and analyzed on ImageJ software.

#### Cytofluorometry

Cells from healthy donors were pelleted by centrifugation after 24 or 72 h exposure to SARS-CoV-2, as described above, before staining for cytoflorometry analysis. PBMC isolated from COVID-19 patients and healthy donors were stained immediately after isolation. Cells were incubated with FcR Blocking Reagent according to manufacturer’s protocol (Miltenyi Biotec, 130-059-901). Cells from healthy donors, used for the exposure to virus *in vitro*, were fixed using Cytofix/Cytoperm kit (BD Biosciences, 51-2090KZ) according to manufacturer’s instructions, before staining. Staining was performed with CD3-PE (BD Biosciences, 552127), CD14-PerCP (BioLegend, 301848), CD19-APC-H7 (BD Biosciences, 560252) and 10 μg/mL GN_mAb_Env01-biotin (GeNeuro, murine antibody) in Perm/Wash buffer (BD Biosciences, 512091KZ). HERV-W ENV expression was revealed using Streptavidin FITC conjugate (eBioscience, 11-4317-87). Background noise was assessed using mouse isotype control (mIgG1-FITC, Miltenyi Biotec 130-113-199) and Streptavidin FITC conjugate (eBioscience, 11-4317-87). Stained cells were acquired on a BD LSR Fortessa (BD Biosciences). Fluorochrome emissions from the pool of antibodies were compensated using Ultra-Comp eBeads plus Compensation beads (Invitrogen, 01-3333-42). Data were analyzed with FlowJo software (v.10).

#### Quantitative RT-PCR (RT-qPCR)

RT-qPCR was performed using specific primers for HERV-W and HERV-K envelope genes, as already validated in patients with HERV-associated diseases,^28^^,^^72^^,^^73^ using B2M mRNA as a suitable reporter gene for PBMC.^74^^,^^75^ For *in vitro* analyses, cells were harvested at several time points after exposure to SARS-CoV-2 virus or protein, and total RNA extracted. For blood samples, freshly isolated PBMC were collected to similarly extract RNA. 200 ng of DNase-treated RNA were reverse-transcribed into cDNA using iScript cDNA Synthesis Kit (Bio-Rad, 1708891) according to the manufacturer’s protocol. A control with no-RT was prepared in parallel, to confirm the absence of contaminating DNA in PCR experiments. An amount of 5 ng of initial RNA in RT reaction has been used to quantitatively evaluate the transcriptional levels of HERV- W *ENV*, HERV-K *ENV*, *N* SARS-CoV-2^76^ and *ACE2* genes by RT-qPCR). The assays were performed in a StepOnePlus instrument (Applied Biosystems) using Platinum SYBR Green (Invitrogen, 11744-500). The housekeeping gene glyceraldehyde-3-phosphate dehydrogenase (GAPDH) was used to normalize results in Vero cells experiments. The RT-qPCR was performed using following primers: HERV-W ENV (forward primer “fwd” [5′- GTATGTCTGATGGGGGTGGAG-3′] and reverse primer “rev” [5‘-CTAGTCCTTTGTAGGGGCTAGAG-3′]; HERV-K ENV fwd [5′-CTGAGGCAATTGCAGGAGTT-3′] and rev [5‘-GCTGTCTCTTCGGAGCTGTT-3′]; N SARS-CoV-2 fwd [5′-AAACATTCCCACCAACAG-3′] and rev [5′- CACTGCTCATGGATTGTT-3′]; ACE2 fwd [5′- TCCATTGGTCTTCTGTCACCCG-3′] and rev [5′- AGACCATCCACCTCCACTTCTC-3′]; B2M fwd [5′- TTACTCACGTCATTCAGCAG-3′] and rev [5‘- GATGGATGAAACCCAGACAC-3′]; GAPDH fwd [5′- CACCCACTCCTCCACCTTTGAC-3′] and rev [5′- AGACCATCCACCTCCACTTCTC-3′]. The conditions of amplification were are presented in the graphical representation below, as generated by the software of StepOnePlus platform.

**Figure:**
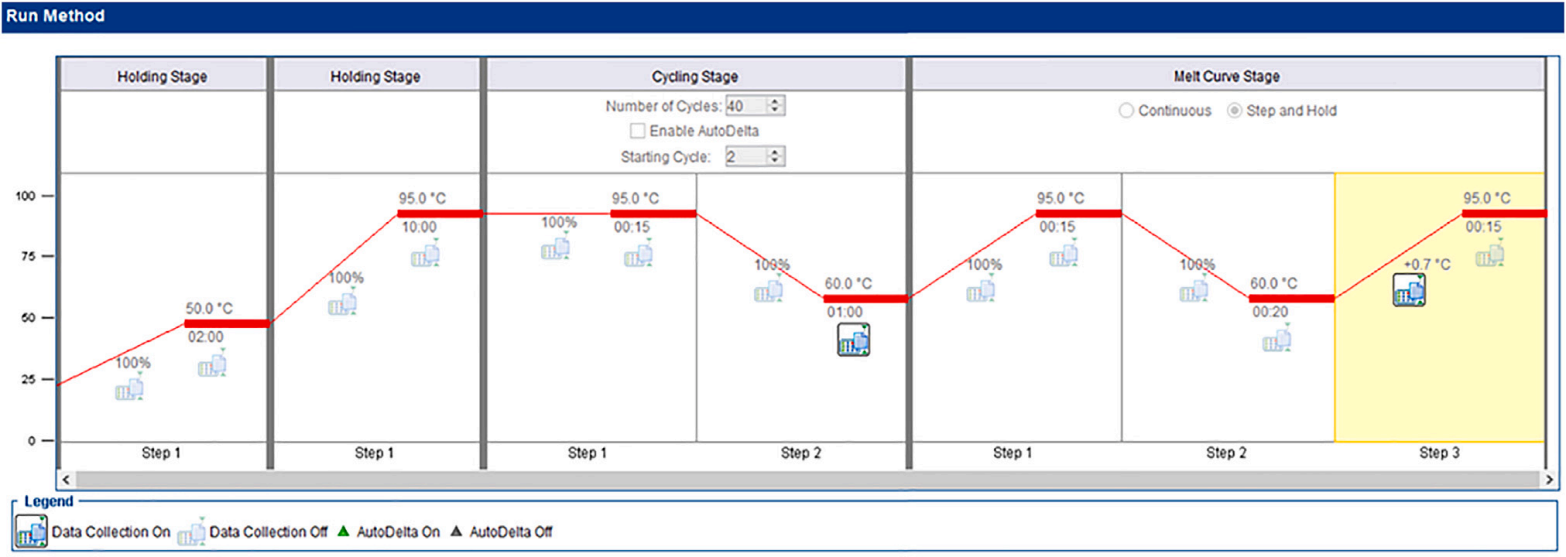

Each experiment was completed with a melting curve analysis to confirm the specificity of amplification and the lack of any non-specific product and primer dimer in presence of the target. Quantification was performed using the threshold cycle (Ct) comparative method: the relative expression was calculated as follow: 2^-[ΔCt (sample) - ΔCt (calibrator)]^ = 2^−ΔΔCt^, where ΔCt (sample) = [Ct (target gene) – Ct (housekeeping gene)] and the ΔCt (calibrator) was the mean of ΔCt of (i) non infected/non treated cells for *in vitro* studies or (ii) PBMC of healthy controls for studies with patients. Details of RT-qPCR results related to cultures of PBMC from HBD in presence of SARS-CoV-2 virus are available at https://doi.org/10.17632/rc74sdgksk.1.

#### Quantification of Interleukin-6 (IL-6) secretion

PBMC, 4x105 cells per well, were cultured in 48 wells plate in presence of RPMI 1640 (Gibco, 61870- 010) completed with 5% of heat inactivated AB human serum (Sigma, H4522). IL-6 secretion was assessed in PBMC culture supernatant 2, 15 and 24 h after recombinant Spike exposure, by ELISA using BD Opt EIA Set Human IL-6 (BD, 555 220) according to supplier’s recommendations. To analyze a possible contribution of endotoxin in the stimulation of IL-6 response, cells were treated during 24 h with a combination of 10 ng/mL LPS-EK (Invitrogen, Tlrl-eklps), 12.5 μg/mL Polymyxin B (inhibitor of endotoxin, Invitrogen, Tlrl-pmb), 2 μg/mL recombinant non-stabilized trimer Spike SARS-CoV-2 (ACROBiosystems, USA; SPN-C52H8) or its buffer in quantities equivalent to 2 μg/mL. IL-6 was measured (triplicates) on culture cell supernatants using the IL-6 ELISA assay kit BD Opt. EIA human IL-6 ELISA set (BD, 555 220) and GloMax plate reader (Promega, GM3000).

#### Cell viability assay

PBMC viability was analyzed using CellTiter-Glo 2.0 Assay kit (Promega, G9241) according to the manufacturer’s protocol. Viability was calculated using the following equation: % of viability = (RLU_experimental_ – RLU_background_)/[Mean (RLU_control_ – RLU_background_)] x 100. (Background: wells containing medium without cells; Control: untreated cells 24h post treatment).

#### Immunocapillary western-blot (Wes) detection of HERV-W ENV and HERV-K ENV in sera or plasma

For the detection of HERV-W ENV antigen, sera or plasma were incubated for 2 h at 25°C with gentle agitation in presence of 1X RIPA buffer (R0278-500 ML, Sigma Aldrich) supplemented with 1% Fos cholin 16 (F316S-1 GM, Anatrace) and protease inhibitor cocktail (5892791001, Roche). For HERV-K ENV antigen, sera or plasma were incubated 30 min on ice in extraction buffer containing 6M urea (GE Healthcare, 17-1319-01), 150 mM NaCl, 20 mM Tris pH 8, 0.6% IGEPAL CA-630 (Sigma, I7771), 2 mM EDTA, 1 mM PMSF and protease inhibitor cocktail (Roche, 0469313001). After 10 min of centrifugation at 10,000 x g, supernatants were collected. Deglycosylation was immediately performed and high molecular weight proteins were enriched on column filter as previously described 15. HERV-W ENV and HERV-K ENV antigen detection was analyzed on the Wes device using SimpleWestern technology an automated capillary-based size sorting and immunolabeling system (ProteinSimpleTM) as previously described (Charvet et al., 2021). The following primary antibodies were used: anti- HERV-W ENV mAb GN_mAb_ENV01 (-W01) (20 μg/mL) and anti- HERV-K ENV mAb GN_mAb_ENV-K01 (30 μg/mL) (GeNeuro). Signal quantification was made by calculating the complete Area Under the Curve (AUC) defined by the curve (peak) above the baseline of the electropherogram, within the apparent molecular weight range of HERV-ENV deglycosylated antigen in this capillary matrix (350–450 KDa), using the Wes platform Compass TM software (ProteinSimple, USA). Because this AUC also accounts for non-specific background signal within capillaries in this high molecular weight range, a cut-off of specificity for the quantification interval corresponding to the protein detection is defined from the background signal generated using initially isotype control mAb and subsequently by the same type of samples from negative healthy controls, to take into account the inter-individual variation that generates this background signal with a given technique and to determine a statistical threshold for specific positivity. The specificity threshold is statistically defined by the mean of negative controls (without possible outliers) + 2 standard deviations (cut-off = mean+2SD of negative controls). A relative quantification is therefore presented as a signal to noise ratio (S/N): Total peak AUC/cut-off = S/N. Positive quantification corresponds to S/N values above 1, and negativity of the detection corresponds to values below 1. Since values below 1 do not represent a quantification of the specific signal due to the antigen, results with S/N < 1 are all similarly negative and therefore normalized to the value S/N = 1. Samples were loaded in triplicates and the global signal containing the target hexamer HERV-W ENV (electrophoregram peak about 440 kDa) or HERV-K ENV protein (electrophoregram peak about 60–90 kDa) was measured. According to standard diagnostic rules for such immunoassays, in case of a single discordant value (CV>15%) was eliminated, keeping two consistent values (CV ≤ 15%) of the triplicate for the calculation of the mean value as a result. In case of three discordant values with CV>15%, the result was deemed not interpretable.

#### Immunohistology (IHC)

Paraffin embedded COVID-19 tissue section slides (from lung, heart, nasal and brain tissue) from 15 patients’ necropsies and 3 non-COVID-19 lung samples (normal appearing tissue from lung cancer) (samples description and clinical data are respectively provided in Figure 7) were rehydrated by successive bath of toluene, degreasing concentration of ethanol and 1X PBS. Depending on the antibody/antigen couple, an antigen retrieval step can be needed, consisting in boiling samples in citrate buffer at an appropriate pH. Endogenous peroxydases were inhibited in a 30 min bath of 4% H2O2. Permeabilization was performed during 5–10 min in 1X PBS+0.2% Tween 20 and the non-specific sites interaction were blocked by an incubation in 3% horse serum in 1X PBS+0.2% Tween 20 during 30 min at RT. Primary antibodies were incubated overnight at 4°C and secondary antibodies were incubated during 45 min at RT. Revelation was performed using AEC kit (Vector, SK-4200) following supplier indications. Nuclei were counter-stained during 3 min with Harris hematoxylin (filtrated and 3-fold diluted) before a quick rinse in water. Slides were mounted using Fluoromount (Southern Biotech). IHC and IF slide observation and image acquisition were performed on NIKON Eclipse TS2R microscope and analyzed using ImageJ software.

### Quantification and statistical analysis

#### Statistics

All statistical analysis was performed using GraphPad Prism 7. For the analysis of the results from cytofluorometry, all measures were performed in triplicates and differences were analyzed using Tukey’s multiple comparisons test (Figure 1D) or unpaired t test and non-parametric Mann-Whitney test (Figures 2F, 3A, 3B, 4A, 4B, and 4E). In Figure 1A, using “F” test was used in order to compare dispersion parameter between SARS-CoV-2-infected PBMC and mock condition. The comparison between HBD and COVID-19 patients grouped by severity of symptoms were assessed using Dunnett’s multiple comparisons test (Figure 4G). The differences in the production of IL-6 were tested using the two-way ANOVA, multiple comparisons Sidak’s test (Figure 2C). The comparison of HERV-W ENV and HERV-K ENV quantification in the serum or plasma of HBD, COVID-19 or other diseases patients were performed using non-parametric Mann- Whitney test (Figures 3C, 4C, and S3A). The heatmap was generated by associating the severity of disease observed in COVID-19 patients with their PBMC cytofluorometric profile based on the detection of membrane positivity for HERV-W ENV (whatever the number of cells). The intensity (heat) of the color-scale, as indicated in the axis, represents the % of patients with positive cells versus those without (negative detection) for each cell subtype in each category of disease severity. Statistical analysis based on symptoms severity, and HERV-W antigenemia were performed using Dunn’s multiple comparisons test (Figure 4D). Statistical significance of differences in RT-qPCR analysis of SARS-CoV-2 N mRNA production was performed using one-way ANOVA, multiple comparisons Sidak’s test (Figure S1E). Differences in the Viability test of PBMC cultures was performed using a two-way ANOVA, multiple comparisons Sidak’s test (Figure S2C). Statistical comparison in the Endotoxin assay was performed using Mann Whitney test (Figure 2D). Finally, all correlations (Figure 3D) were assessed by Pearson “r” test using the following scoring based on Pearson’s table r = 0, no correlation; 0 < r < 0.2, weak correlation; 0.2 < r < 0.5, mean correlation; 0.2 < r < 0.5, mean correlation; 0.5 < r < 0.8, strong correlation; r > 0.8, strong correlation; r = 1 perfect correlation. Significance of all observed differences was presented by the “p” value (∗p < 0.05, ∗∗p < 0.01, ∗∗∗p < 0.001 and ∗∗∗∗p < 0.0001).

## Data Availability

• Clinical data of patients cohort have been deposited at Mendeley data website. • This paper does not report original code.
