# Supplementary material for: SARS-CoV-2 awakens ancient retroviral genes and the expression of proinflammatory HERV-W envelope protein in COVID-19 patients
Source: iScience. 2023 Apr 7;26(5):106604. doi: 10.1016/j.isci.2023.106604 (PMC10079620; doi:10.1016/j.isci.2023.106604)
Supplement: Document S1. Figures S1–S4 [file mmc1.pdf]

iScience, Volume *26*

Supplemental information

SARS-CoV-2 awakens ancient retroviral genes and the expression of proinflammatory HERV-W envelope protein in COVID-19 patients

Benjamin Charvet, Joanna Brunel, Justine Pierquin, Mathieu Iampietro, Didier Decimo, Nelly Queruel, Alexandre Lucas, María del Mar Encabo-Berzosa, Izaskun Arenaz, Tania Perez Marmolejo, Arturo Ivan Gonzalez, Armando Castorena Maldonado, Cyrille Mathieu, Patrick Küry, Jose Flores-Rivera, Fernanda Torres-Ruiz, Santiago Avila-Rios, Gonzalo Salgado Montes de Oca, Jon Schoorlemmer, Hervé Perron, and Branka Horvat

Supplemental information:

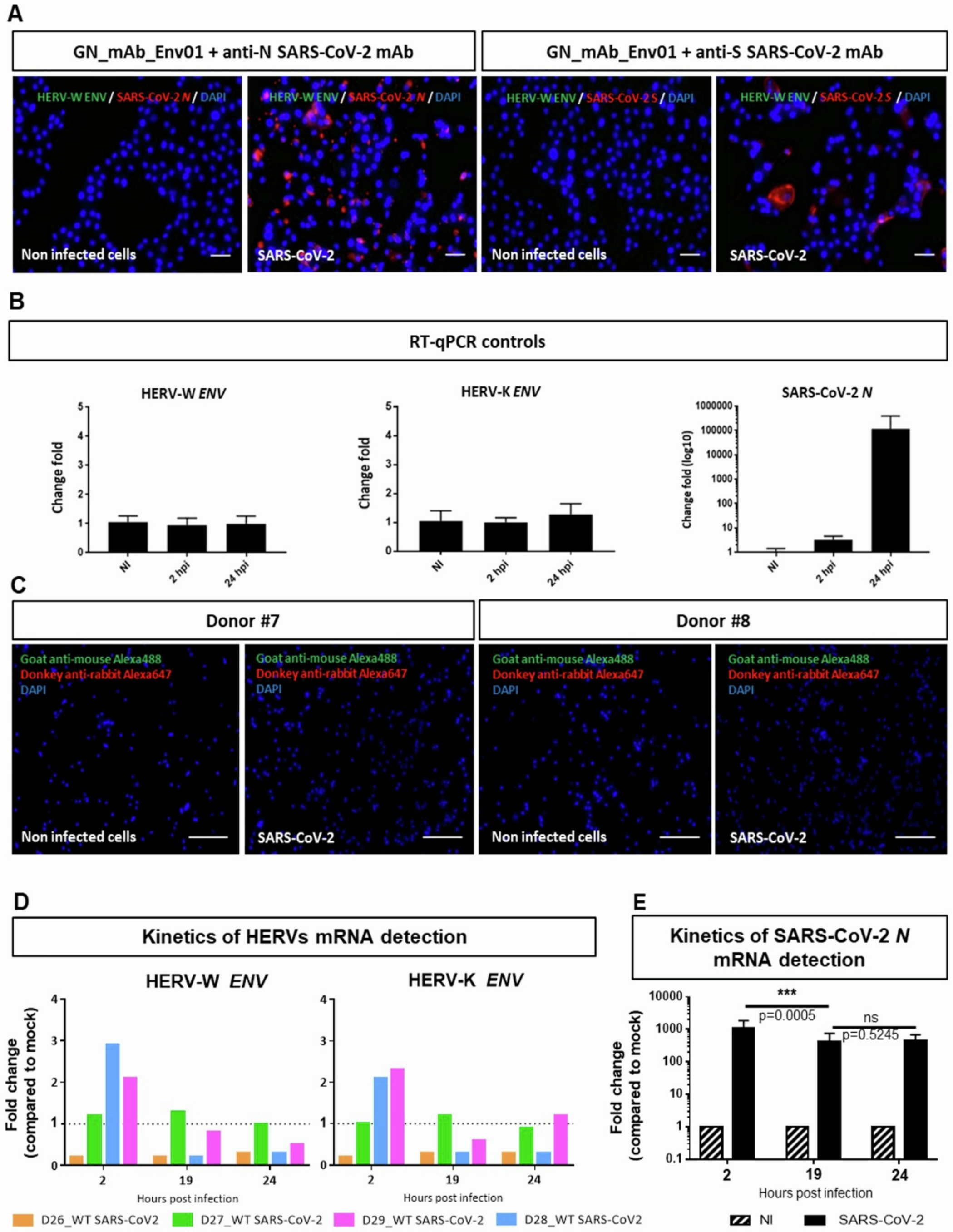

Figure S1 related to Fig 1. Kinetics of HERVs and SARS-CoV-2 mRNA expression during PBMC infection and controls for antibody staining. A. Confirmation of the specificity of anti-HERV-W (GN_mAb_Env01) and anti-SARS-CoV-2 mAbs, used for immunostaining. Non-infected Vero cells were used as negative controls for immunostaining, while SARS-CoV-2 infected Vero cells were used as positive controls for anti-N- and anti-SARS-CoV-2 -S immunodetection (red staining). Vero cells do not express HERV-W ENV protein (green staining). DAPI was used to stain nuclei (blue staining). Bars = 50 µm. **B.** Vero cells showed no expression of HERV-W *ENV* and HERV-K *ENV* mRNA at 2h pi (hours post-infection) and 24h pi. The efficiency of viral replication was monitored between 2h and 24h pi by RT-qPCR, using N-SARS-CoV-2 primers. RT-qPCR results were presented as the fold change compared to the corresponding non-infected condition (NI). **C.** Background staining induced by goat anti-mouse-Alexa488 and Donkey anti-rabbit-Alexa647 secondary antibodies was evaluated using negative controls in the absence of primary antibodies, with PBMC cultures from 2 healthy donors (donors #7, 8), exposed for 72 h to the infectious SARS-CoV-2 virus, at MOI:0.1, or sham-inoculated. DAPI was used to stain nuclei (blue staining). Bars = 50 µm. **D**. Kinetics of HERV-W *ENV* and HERV-K *ENV* mRNA in PBMC from healthy blood donors exposed *in vitro* to SARS-CoV-2. PBMCs from 4 healthy blood donors (D26 to D28) were exposed to SARS-CoV-2 virus (MOI: 0.1) in parallel to mock-control (medium without virus). HERV-W *ENV* and HERV-K *ENV* mRNA levels were assessed at 2h, 19h and 24h post-inoculation (pi) by RT-qPCR and results are presented as the fold change from the corresponding non-infected condition. **E.** The mRNA levels of SARS-CoV-2 *N* in PBMCs cultures from the same 4 healthy blood donors, exposed (plain histograms) or not (hatched histograms) to infectious SARS-CoV-2 (MOI:0.1), were analyzed by RT-qPCR. Results are presented as RT-qPCR fold change compared to the corresponding non-infected condition. NI: non-infected control culture; pi: post-inoculation. The quantity of SARS-CoV-2 N mRNA was monitored 2h, 19 h and 24h after SARS-CoV-2 infection (black histograms).

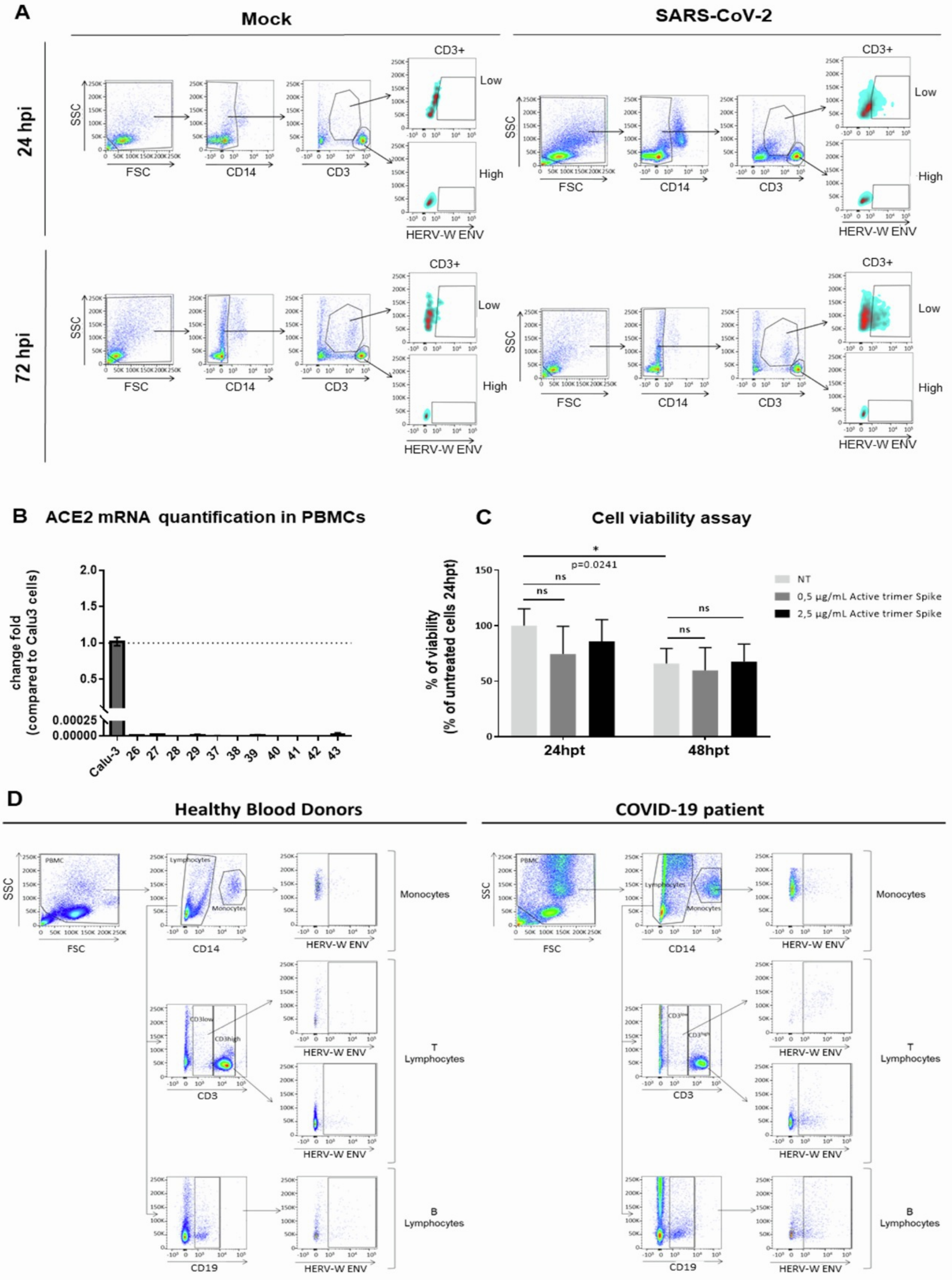

Figure S2 related to Fig. 1 to Fig. 4. Gating strategy for cytofluorometry studies and control analysis of PBMC cultures. A. Examples of gating strategy for HERV-W ENV detection using cytofluorometry in PBMCs subpopulation after exposure to SARS-CoV-2 virus. PBMCs from healthy donors were either incubated with SARS-CoV-2 (MOI=0.1) for 24h or 72h, or remained unexposed to the virus. Cells were stained for membrane CD3 and CD14 and intracellular HERV-W ENV expression and analyzed by flow cytometry. The percentage of HERV-W ENV positive cells in CD14 and CD3 T cells, with identification of CD3^{high} and CD3^{low} T cell subpopulations (after suppression of CD14 cells from the gating) was determined. **B.** RT-qPCR with specific ACE2 primers in PBMCs from 11 healthy blood donors (HBD), showing the absence of detectable mRNA expression in human PBMC. **C.** PBMCs isolated from 5 healthy blood donors (donors #17 to 21), were treated, or not (NT), with 0.5 µg/mL or 2.5 µg/mL of active trimer Spike recombinant protein. Cell viability in cultures was assessed using the “CellTiter-Glo 2.0®” kit assay. Results were expressed as the percentage of viability measured in the non-treated culture at 24 h post treatment (hpt). **D.** Examples illustrating the gating strategy for cytofluorometry analysis performed on cohort COVID-19 patients and controls PBMC. Dot plots of a representative HBD (left panel) and a COVID-19 patient (right panel). This allowed to determine the percentage of membrane HERV-W ENV positive cells in CD14 and CD3 T cell populations, with identification of CD3^{high} and CD3^{low} T cell subpopulations, as well as of HERV-W ENV positive cells in CD19^{+} B cells population. Of note, the specific identification of CD3^{low} T cells with an increased size was confirmed with the suppression of CD14 positive monocyte cells from the gating. The analysis was done by acquiring at least 50 000 events in the PBMC gate.

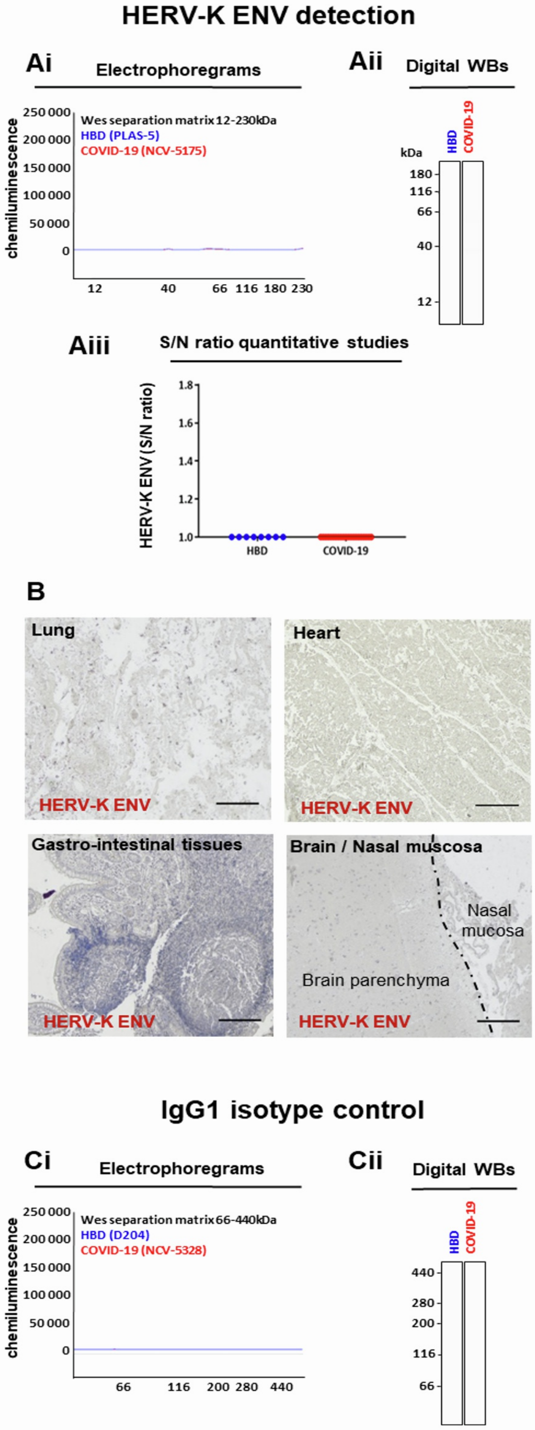

Figure S3 related to Fig. 3, Fig. 5 and Fig. 6. Absence of HERV-K ENV protein detection in plasma and in post-mortem tissues from COVID-19 patients and absence of HERV-W ENV protein detection with the isotype control antibody. **A.** Analysis of the presence of HERV-K ENV in plasma from HBD and COVID-19 patients. The electrophoregrams failed to detect a specific peak in the 60-90 KDa region, as expected from the variously glycosylated forms of the HERV-K ENV protein (Ai), which was also illustrated by the digital WB presentation (Aii). The same series of HBD and patients' plasma as in Figure 3C (see cohort "Lyon" details; DOI: 10.17632/3v4hfxv4w8.1) for HERV-W, revealed all negative (Aiii). **B.** Tissue samples of different organs from autopsied COVID-19 cases, were also analyzed with a specific anti-HERV-K mAb targeting a highly conserved epitope. Sections were selected for SARS-CoV-2 and HERV-W ENV positive areas (cf. next figures) and all showed an absence of immunostaining in lung, heart, digestive tract, illustrated with intestinal epithelium and mucosa associated lymphoid tissue and the interface of brain parenchyma with olfactory mucosa across the cribriform plate, indicated by a dotted line. **C.** In order to evaluate a potential non-specific signal generated by the primary antibody, an IgG1 isotype control was applied on plasma from COVID-19 patients (red panel) or HBD (blue panel). (Ci) No detectable signal was observed on a 66-440 kDa separation matrix (suitable for detection of HERV-W ENV hexamer). This absence of signal recorded on electrophoregrams resulted in the absence of a visible band on the digital Western Blot representation (Cii).

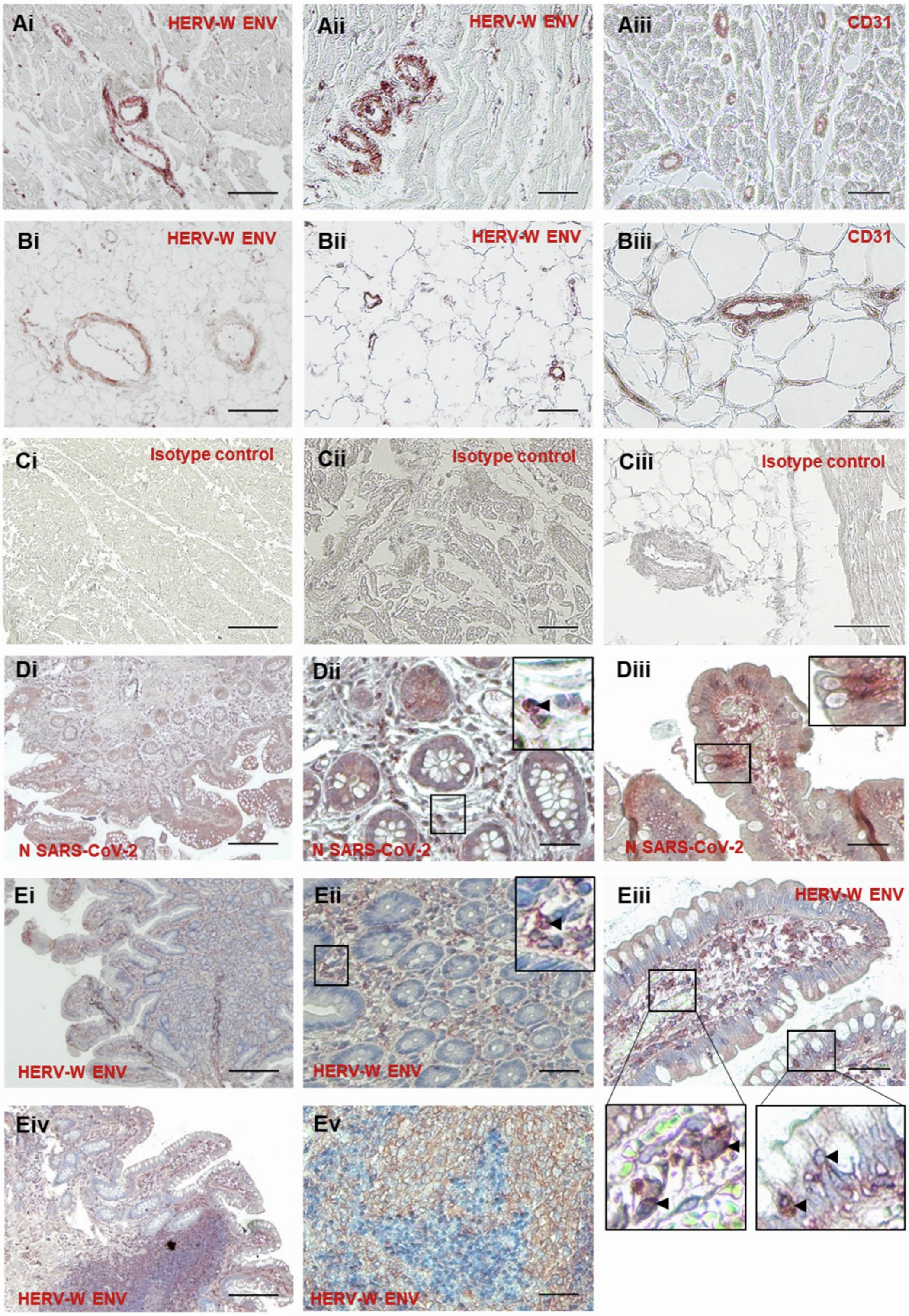

Figure S4 related to Fig. 7. Immunohistology detection of HERV-W ENV protein in post-mortem cardiac and gastrointestinal tissues from acute COVID-19 patients. A. Sections of post-mortem cardiac muscle from COVID-19 patients were immunostained with anti-HERV-W ENV (Ai and Aii) and anti-CD31 (Aiii) as specific marker of endothelial cells confirming the nature of HERV-W positive structures (brown/red staining). Bars = 100 µm. **B.** On the same sections, pericardia fatty tissue was also observed for HERV-W ENV (Bi and Bii) and CD31 (Biii) immunostaining. Bars = 100 µm. **C.** Murine IgG1 isotype control antibodies was tested on adjacent sections of cardiac muscle (Ci and Cii) and pericardia fatty tissue (Ciii) and did not show unspecific background. Bars = 100 µm. **D.** Sections of digestive tract tissues from COVID-19 patients were stained for N-SARS-CoV-2 (Di-Diii) (brown-red staining). As shown with sections from terminal ileum, N-SARS-CoV-2 was detected in intestinal epithelial cells (Di), in sub-mucosal cells and glands (Dii) and appeared to be expressed in the cytoplasm of few goblet cells at the epithelial level (Diii). Di : Bars = 250 µm ; Dii and Diii : Bars = 100 µm. **E.** Sections of digestive tract tissues from COVID-19 patients were also stained for HERV-W ENV (Ei – Ev) (brown-red staining). HERV-W ENV protein was mainly detected in lymphoid-shaped cells infiltrated in mucosal and sub-mucosal areas (Ei-Eiii) with few positive macrophage-like cells found below (Eiii, top left magnification) or inserted between (Eiii, bottom right magnification) epithelial cells. Numerous lymphoid cells were HERV-W ENV positive in mucosa associated lymphoid tissue (Eiv and Ev). Ei and Eiv : Bars = 250 µm ; Eii, Eiii and Ev: Bars = 100 µm.
